# Root branching under high salinity requires auxin-independent modulation of LATERAL ORGAN BOUNDARY DOMAIN 16 function

**DOI:** 10.1093/plcell/koad317

**Published:** 2023-12-23

**Authors:** Yanxia Zhang, Yiyun Li, Thijs de Zeeuw, Kilian Duijts, Dorota Kawa, Jasper Lamers, Kristina S Munzert, Hongfei Li, Yutao Zou, A Jessica Meyer, Jinxuan Yan, Francel Verstappen, Yixuan Wang, Tom Gijsberts, Jielin Wang, Nora Gigli-Bisceglia, Timo Engelsdorf, Aalt D J van Dijk, Christa Testerink

**Affiliations:** Laboratory of Plant Physiology, Wageningen University & Research, 6708 PB Wageningen, The Netherlands; Plant Cell Biology, Faculty of Science, Swammerdam Institute for Life Sciences, University of Amsterdam, 1090 GE Amsterdam, The Netherlands; College of Agriculture, South China Agricultural University, 510642 Guangzhou, China; Laboratory of Plant Physiology, Wageningen University & Research, 6708 PB Wageningen, The Netherlands; Laboratory of Plant Physiology, Wageningen University & Research, 6708 PB Wageningen, The Netherlands; Laboratory of Plant Physiology, Wageningen University & Research, 6708 PB Wageningen, The Netherlands; Plant Cell Biology, Faculty of Science, Swammerdam Institute for Life Sciences, University of Amsterdam, 1090 GE Amsterdam, The Netherlands; Laboratory of Plant Physiology, Wageningen University & Research, 6708 PB Wageningen, The Netherlands; Molecular Plant Physiology, Philipps-Universität Marburg, 35043 Marburg, Germany; Laboratory of Plant Physiology, Wageningen University & Research, 6708 PB Wageningen, The Netherlands; Laboratory of Plant Physiology, Wageningen University & Research, 6708 PB Wageningen, The Netherlands; Laboratory of Plant Physiology, Wageningen University & Research, 6708 PB Wageningen, The Netherlands; Laboratory of Plant Physiology, Wageningen University & Research, 6708 PB Wageningen, The Netherlands; Laboratory of Plant Physiology, Wageningen University & Research, 6708 PB Wageningen, The Netherlands; Laboratory of Plant Physiology, Wageningen University & Research, 6708 PB Wageningen, The Netherlands; Laboratory of Plant Physiology, Wageningen University & Research, 6708 PB Wageningen, The Netherlands; Laboratory of Plant Physiology, Wageningen University & Research, 6708 PB Wageningen, The Netherlands; Laboratory of Plant Physiology, Wageningen University & Research, 6708 PB Wageningen, The Netherlands; Molecular Plant Physiology, Philipps-Universität Marburg, 35043 Marburg, Germany; Bioinformatics Group, Wageningen University & Research, 6708 PB Wageningen, The Netherlands; Laboratory of Plant Physiology, Wageningen University & Research, 6708 PB Wageningen, The Netherlands

## Abstract

Salinity stress constrains lateral root (LR) growth and severely affects plant growth. Auxin signaling regulates LR formation, but the molecular mechanism by which salinity affects root auxin signaling and whether salt induces other pathways that regulate LR development remains unknown. In *Arabidopsis thaliana*, the auxin-regulated transcription factor LATERAL ORGAN BOUNDARY DOMAIN 16 (LBD16) is an essential player in LR development under control conditions. Here, we show that under high-salt conditions, an alternative pathway regulates *LBD16* expression. Salt represses auxin signaling but, in parallel, activates ZINC FINGER OF ARABIDOPSIS THALIANA 6 (ZAT6), a transcriptional activator of *LBD16*. ZAT6 activates *LBD16* expression, thus contributing to downstream cell wall remodeling and promoting LR development under high-salt conditions. Our study thus shows that the integration of auxin-dependent repressive and salt-activated auxin-independent pathways converging on LBD16 modulates root branching under high-salt conditions.

## Teaser

Salt represses auxin signaling but, in parallel, activates a transcriptional cascade that is required for root branching.

**IN A NUTSHELL**

**Background:** Soil salinity causes crop yield losses worldwide. Adjusting plant root growth and development is crucial for plants to cope with salt stress. Lateral roots that branch out from the main roots are important to allow plants to absorb water and nutrients in saline soils. Lateral root development is generally controlled by plant hormones, and auxin is a major hormone regulating the formation of lateral roots. In the model plant species Arabidopsis (*Arabidopsis thaliana*), the auxin pathway-regulated transcription factor LATERAL ORGAN BOUNDARY DOMAIN 16 (LBD16) was reported to act downstream of AUXIN RESPONSE FACTOR (ARF)7 and ARF19 to promote lateral root development.

**Question:** How is auxin signaling affected by salinity in roots and are there alternative components that contribute to lateral root development during salt stress?

**Findings:** We discover that salt has a negative effect on auxin signaling in different regions along Arabidopsis main roots, whereas the gene expression of *LBD16* is enhanced by salt. In addition, *lbd16* mutants have reduced lateral root densities along the main roots in high-salt conditions. Therefore, *LBD16* is required for lateral root development in salt, but might be regulated by additional upstream factors besides auxin. Further bioinformatic prediction of gene regulatory networks and experimental validation reveal that salt activates the transcription factor ZINC FINGER OF ARABIDOPSIS THALIANA 6 (ZAT6) independently from ARF7 and ARF19 to modulate the activity of *LBD16*. This salt-induced module governs downstream cell wall remodeling and promotes root branching.

**Next steps:** Salt stress has overall negative effects on auxin-mediated growth regulatory pathways. However, our study demonstrates that salt activates alternative (positive) pathways to contribute to root growth regulation. We need to understand how these auxin-dependent repressive and salt-activated auxin-independent pathways interact to understand how root growth is regulated under salinity.

## Introduction

Plant root system architecture (RSA) has important effects on plant survival and productivity in response to environmental challenges. In Arabidopsis (*Arabidopsis thaliana*), lateral roots (LRs) contribute to the majority of the mature root system. LR formation and growth are tightly regulated by internal chemical signals and external environmental cues, for example soil salinity and water availability (Julkowska et al. 2017; Orosa-Puente et al. 2018). LRs are specified within the main root oscillation zone, from the meristem zone to the elongation zone, and initiate from xylem pole pericycle (XPP) cells in the differentiation zone (Du and Scheres 2018). During LR initiation, asymmetric cell division takes place and results in the formation of Stage I lateral root primordia (LRP) in the early differentiation zone, which is followed by early morphogenesis (Stages II through IV) and further meristem organization (Stages V to VIII) of the LRP to traverse the overlaying cell layers and establish emerged LRs (Malamy and Benfey 1997; Du and Scheres 2018; Banda et al. 2019).

Cell wall changes occur in the endodermal cell layer overlaying the LRP in maize (*Zea mays*) roots, a phenomenon documented as early as in the 1970s (Bell and McCully 1970). The esterification state of pectin at LR initiation sites affects LR formation in Arabidopsis and results from the action of several PECTIN METHYLESTERASEs (PMEs) (PME2, PME3, and PME5) and PME INHIBITOR 3 (PMEI3) (Wachsman et al. 2020). Additionally, the cell wall–loosening proteins EXPANSIN A1 (EXPA1), EXPA14, and EXPA17 have also been reported to promote LR formation (Lee et al. 2013; Lee and Kim 2013; Ramakrishna et al. 2019). Thus, LR development requires coordination of cell division and cell wall modifications to accommodate the emerging LRs.

The plant hormone auxin plays indispensable roles during various stages of LRP development from initiation to LR emergence via different AUXIN RESPONSE FACTOR (ARF)–mediated transcriptional modules (Lavenus et al. 2013; Santos Teixeira and Ten Tusscher 2019). Arabidopsis ARF7 and ARF19 mediate LR development by transcriptional regulation of several redundant *LATERAL ORGAN BOUNDARY DOMAIN* (*LOB*) family transcription factor genes including *LBD16*, *LBD18*, *LBD29*, and *LBD33* (Okushima et al. 2007; Goh et al. 2012; Porco et al. 2016). Auxin was shown to be upstream of the cell wall remodeling pathway(s) during LR development. For example, auxin induces INFLORESCENCE DEFICIENT IN ABSCISSION (IDA)–HAESA (HAE)/HAESA-LIKE 2 (HSL2) ligand–receptor signaling to activate the mitogen-activated protein kinase (MAPK) cascade MAPK KINASE 4/5 (MKK4/MKK5)–MPK3/MPK6, which is required for the expression of cell wall remodeling genes during LR emergence (Kumpf et al. 2013; Zhu et al. 2019). The enzymes XYLOGLUCAN ENDOTRANSGLUCOSYLASE 9 (XTH9) and XTH23 are involved in the endotransglucosylation and endohydrolysis of the major cell wall hemicellulose xyloglucan and act downstream of auxin signaling to mediate LR development (Kumpf et al. 2013; Xu and Cai 2019). In summary, auxin, cell wall modification, and their interplay all play essential roles in mediating LR development. However, it is not clear how auxin signaling and cell wall modifications are modulated during LR development in response to external environmental signals.

In response to high salinity, Arabidopsis plants remodel their RSA by modulating LR emergence and elongation (Zolla et al. 2010; Julkowska et al. 2014; Kawa et al. 2016; Julkowska et al. 2017), resulting in shorter and fewer LRs in general, although natural variation in the response has been observed. Genetic components contributing to LR developmental plasticity in response to salinity in Arabidopsis have been identified, including the *SALT OVERLY SENSITIVE* (*SOS*) pathway gene *SOS3* (Zhao et al. 2011), *HIGH-AFFINITY K^+^ TRANSPORTER 1* (*HKT1*), *CYTOCHROME P450 FAMILY 79 SUBFAMILY B2* (*CYP79B2*) (Julkowska et al. 2017), and the WRKY transcription factor gene *WRKY46* (Ding et al. 2015), encoding proteins involved in ion homeostasis, auxin-related metabolite biosynthesis, and regulation of phytohormone homeostasis, respectively. Nevertheless, these studies cannot explain how auxin signals are affected during LR development in response to salinity, and how salt affects the core LR developmental pathways is largely unknown.

Here, we report that LBD16 is a crucial mediator of LR developmental plasticity in high-salt conditions, enabling cell wall remodeling. We show that salt upregulates *LBD16* expression in the main root zones and developing LRP independently of ARF7 and ARF19. Simultaneously, we observed a decrease in root auxin levels upon salt treatment, revealing the presence of an alternative auxin-independent pathway promoting *LBD16* upregulation by salt stress. Yeast 1-hybrid (Y1H) screening combined with transcriptomic analysis and network inference identified additional potential salt stress–induced upstream transcriptional regulators of *LBD16*. We predicted that the C2H2-type transcription factor ZINC FINGER OF ARABIDOPSIS THALIANA 6 (ZAT6) plays a pivotal role in activating *LBD16* transcription in response to salt and confirmed that ZAT6 binds to the *LBD16* promoter, acting independently of ARF7 and ARF19 to positively regulate *LBD16* expression, contributing to cell wall modifications in roots in response to salt. Loss-of-function mutants of *ZAT6* displayed salt stress–induced defects in LR development and cell wall composition, similar to the *lbd16-1* mutant. Our study thus provides molecular insights into the coordination of LR developmental plasticity mediated by a salt-activated pathway that positively affects root branching.

## Results

### LBD16 is a central regulator of the plasticity of LR development in response to high-salt conditions

**Figure 1.**
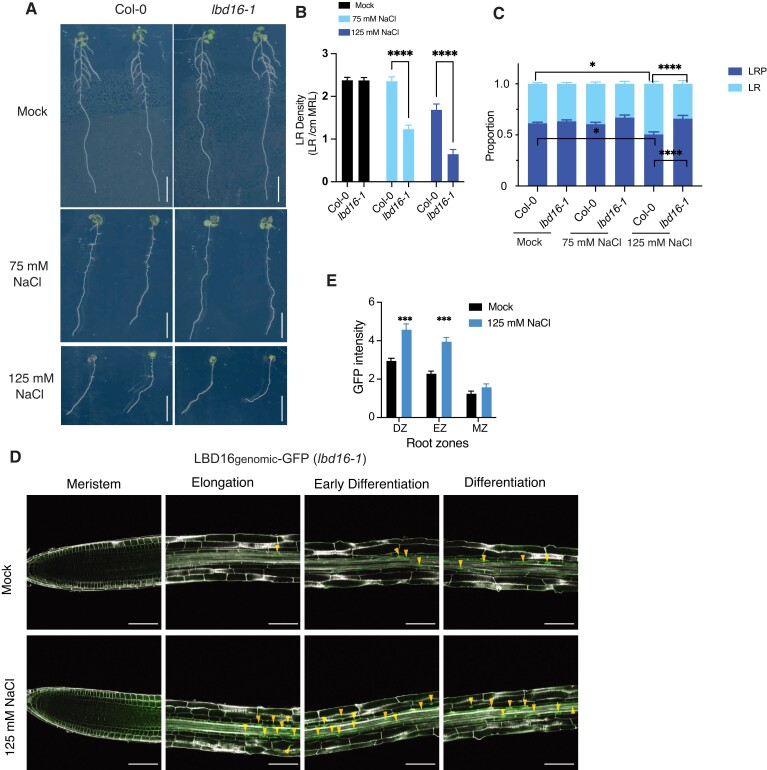
Identification of LBD16 as a mediator of LR development in response to salt. **A to C)** Phenotypic analysis of emerged LRs and nonemerged LRP in Col-0 and *lbd16-1* under mock (0 mM) and salt conditions (75 or 125 mM NaCl). **A)** Representative photographs of roots from 10-d-old Col-0 and *lbd16-1* seedlings in mock and salt conditions. Scale bars, 1 cm. **B)** Density of emerged LRs in Col-0 and *lbd16-1* in mock and salt conditions (*n* = 20 to 25). MRL, main root length. **C)** Distribution of LRs and LRP in Col-0 and *lbd16-1* in mock and salt conditions. Data represent a pool of 4 independent experiments (*n* = 7 to 13 in each experiment). **D)** GFP fluorescence pattern from the *LBD16genomic-GFP* construct in root zones after mock treatment (0 mM NaCl) or treatment with 125 mM NaCl for 6 h of 6-d-old seedlings. *LBD16genomic*-GFP signals are highlighted with arrows. Scale bars, 100 *µ*m. **E)** Quantification of GFP fluorescence intensity from the *LBD16genomic-GFP* transgenic line in root zones (*n* = 22 to 23). Data in **B)**, **C)**, and **E)** represent means ± standard error of the mean (SEM). Statistical analysis in **B)** was done using 2-way ANOVA followed by Šídák’s multiple comparisons test. Statistical analysis in **C)** was done in R by fitting a generalized linear model. Statistical analysis in **E)** was done using a *t* test. *P < 0.05, ***P < 0.001, and ****P < 0.0001. Data in **A to E)** represent multiple independent experiments.

RSA analysis of an Arabidopsis haplotype map (HapMap) natural diversity panel in response to salt stress discovered the previously identified auxin-dependent transcription factor *LBD16* as a candidate locus regulating RSA remodeling in response to salt stress (Julkowska et al. 2017). Since LBD16 had been shown to regulate LR development (Okushima et al. 2007; Goh et al. 2012), we further characterized the RSA of knockout and complementation lines of *LBD16* under control (0 mM NaCl) and various salt conditions using an agar plate setup. We determined that the decrease in the number of emerged LRs of 2 independent *lbd16* mutant alleles (*lbd16-1* and *lbd16-2*) in response to salt is more severe compared to that of the wild-type Col-0 (Fig. 1, A and B; Supplementary Fig. S1). This lower LR density was not due to main root length differences between Col-0 and the mutants (Supplementary Fig. S2A), and using a previously generated complementation line *LBD16genomic*-GFP (Goh et al. 2012), with a wild-type genomic region of the *LBD16* locus in the *lbd16-1* mutant, we could rescue the profound decrease in emerged LR density under both mild (75 mM NaCl) and severe (125 mM NaCl) salt stress conditions back to wild-type values (Supplementary Fig. S2B). We observed no significant decrease in LR density under control conditions in the *lbd16* knockout mutants compared to Col-0 (Fig. 1, A and B; Supplementary Fig. S2B), which is in agreement with previous reports showing the functional redundancy of LBD16 with LBD18 and LBD33 under optimal growth conditions with regard to LR development (Lee et al. 2009; Goh et al. 2012).

To understand how LBD16 may act under saline conditions, we used microscopy to examine LRP development in Col-0 and *lbd16-1* in response to mild and severe salt stress. In line with the reported effect of the *lbd16-1* mutant on LR density (Goh et al. 2012), we also observed a lower density of nonemerged LRP (Stages I through VII) in *lbd16-1* than in Col-0 under control conditions (Supplementary Fig. S2C). Salt stress hampered LR emergence in Col-0, as we counted more nonemerged primordia under 125 mM NaCl than under control conditions (Fig. 1C). Compared to Col-0 roots, *lbd16-1* roots displayed a significantly lower proportion and density of emerged LRs under both 75 and 125 mM NaCl treatments (Fig. 1C; Supplementary Fig. S2C), suggesting that LBD16 is a positive regulator of LRP emergence under high-salt conditions.

We analyzed the dynamic expression patterns of *LBD16* in mature root systems of 4-wk-old hydroponically grown Arabidopsis Col-0 wild-type plants after 3, 6, 12, 18, and 24 h treatment with 125 mM NaCl: we observed that *LBD16* expression is enhanced after 3 h and reaches a peak at 6 h into salt treatment (Supplementary Fig. S3A). In addition, we generated a promoter reporter line harboring the *GUS* reporter gene driven by the *LBD16* promoter (*LBD16pro:GUS*). Seedlings showed higher *LBD16* promoter activity at 6 h into salt treatment with 125 mM NaCl in different regions of the primary root (Supplementary Fig. S3B), from the elongation zone through the differentiation zone, and up to the LR zone where LRs emerge (Supplementary Fig. S3C). In parallel, the *lbd16-1 LBD16_genomic_-GFP* complementation line also showed higher *LBD16* expression in the main root elongation and differentiation zones by treatment with 125 mM NaCl (Fig. 1, D and E). Salt-induced *LBD16*-expressing cell types were not limited to the root stele but also included the endodermis and cortex layers overlaying the developing LRP in the differentiation zone and the LR zone (Supplementary Fig. S3, B and C), suggesting that LBD16 might be involved in LRP development from early phase initiation to LR emergence passing through various root cell layers under salt conditions. Taken together, salt may positively activate an LBD16-mediated pathway in main root zones, which is required for the plasticity of LR development.

### Salt activates an auxin-independent pathway mediating *LBD16* expression

**Figure 2.**
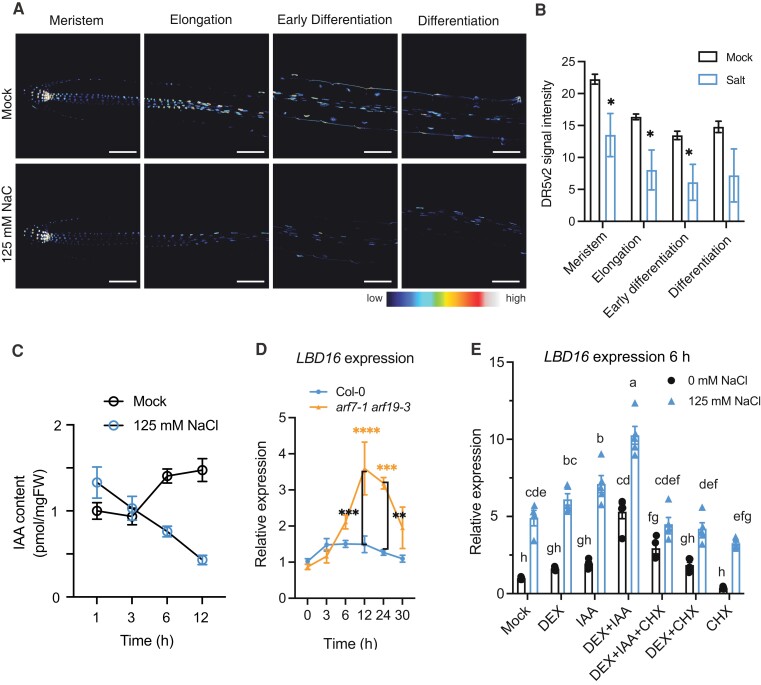
Salt activates an auxin-independent pathway mediating *LBD16* expression. **A)** Auxin output signals as indicated by the *DR5v2:n3mTurqoise2* reporter in main root zones. **B)** Quantification of *DR5v2:n3mTurqoise2* signal intensity in root zones of 6-d-old seedlings encompassing the meristem, elongation zone, and (early) differentiation zone. **C)** Relative IAA content normalized by sample weight and internal standard (^13^C_6_-IAA) in the roots of 7-d-old Col-0 seedlings by LC-MS/MS after mock treatment (0 mM) or 125 mM NaCl treatment for 1, 3, 6, or 12 h (*n* = 4, Supplementary Data Set 5). **D)** Relative *LBD16* expression in the roots of 7-d-old Col-0 and *arf7-1 arf19-3* seedlings after treatment with 125 mM NaCl for 3, 6, 12, 24, or 30 h (*n* = 4). **E)** Relative *LBD16* expression in *arf7-1 arf19-1 ARF7pro:ARF7-GR* plants treated with 1 *µ*M IAA, 2 *µ*M DEX, and/or 10 *µ*M CHX in control (0 mM) or salt conditions (6 h of treatment with 125 mM NaCl) in comparison to mock (no supplementation of DEX, IAA, or CHX) treatment (Supplementary Data Set 5). Expression values were fold changes normalized by mock treatment under 0 mM. The housekeeping gene At2g43770 was used for normalization in **D)** and **E)**. Statistical analyses in **B)** and **C)** were done using a 2-sided *t* test. Statistical analyses in **D)** and **E)** were done using 2-way ANOVA followed by Tukey's multiple comparisons test. *P < 0.05, **P < 0.01, ***P < 0.001, and ****P < 0.0001. Lowercase letters (a to h) above the bars in **E)** indicate the statistical differences, and data with the same letter are not significantly different.

Since *LBD16* was previously shown to be involved in auxin-regulated LR development (Okushima et al. 2007), we asked whether salt would affect auxin signaling during LR development. We used a high-resolution C3PO auxin reporter line that contains a 3-color reporter by incorporating a *DR5v2:n3mTurqoise2* (auxin output readout) cassette into the construct carrying the R2D2 (auxin input) cassette (*DR5v2:mTurquoise-NLS*, *RPS5apro:mDII-ntdTomato*, and *RPS5apro:DII-n3xVenus*) to visualize auxin dynamics at a cellular level during LR development under salt stress (Küpers et al. 2023). We determined that DR5v2 activity is lower in the root zones consisting of meristem, elongation, and early differentiation zones at 6 h into treating seedlings with 125 mM NaCl (Fig. 2, A and B), in accordance with decreased indole-3-acetic acid (IAA) levels in Col-0 roots measured by LC-MS/MS at 6 h of 125 mM NaCl treatment (Fig. 2C). In the LRP, we observed a significant decrease in the auxin input R2D2 signal (indicated by the mDII/DII ratio) in Stages I and II 6 h into treatment with 125 mM NaCl, while we did not detect significant changes in the auxin output signal (*DR5v2*) in developing early stage LRP in response to salt (Supplementary Fig. S4, A and B). Although these results are in line with salt inhibiting LR formation in Col-0 (Fig. 1C; Supplementary Fig. S2C), they do raise the question how salt enhances *LBD16* expression in roots (Fig. 1, D and F) and whether this would occur independently of auxin.

As *LBD16* expression in control conditions is induced by ARF7 and ARF19 acting downstream of auxin, we evaluated *LBD16* expression in the *arf7-1 arf19-3* double knockout mutant. Interestingly, we observed that salt treatment induces an increase in *LBD16* expression in the roots for both the *arf7-1 arf19-3* double mutant and Col-0 seedling, reaching higher levels in the *arf7-1 arf19-3* background (Fig. 2D). This result suggests the existence of an ARF7/ARF19 independent pathway under salt conditions to regulate *LBD16* expression. Since ARF7 was reported to be able to bind to the *LBD16* promoter region and regulate its expression (Okushima et al. 2007), to further investigate the regulation of *LBD16* by ARF7 under salt stress, we made use of a *arf7-1 arf19-1 ARF7pro:ARF7-GR* transgenic line with dexamethasone (DEX)-inducible ARF7 activity (Okushima et al. 2007). In line with a previous study (Okushima et al. 2007), *LBD16* expression was induced by DEX treatment together with IAA in the roots of *arf7-1 arf19-1 proARF7:ARF7-GR* seedlings in control conditions, and this high expression was maintained by the combination of IAA and DEX in the presence of the protein synthesis inhibitor cycloheximide (CHX) to inhibit production of new ARF7 protein (Fig. 2E), confirming that LBD16 is a primary target of ARF7 in control conditions. In response to salt stress, *LBD16* expression was induced significantly by the combination of DEX and IAA in the presence of CHX already early at 3 h of treatment when compared to treatments with DEX and IAA alone (Supplementary Fig. S4C), indicating that LBD16 is primarily regulated by auxin–ARF7 signaling during early salt response at 3 h. However, we observed that the presence of CHX together with DEX and IAA to induce ARF7 activity does not promote *LBD16* expression at 6 h of salt treatment, and that *LBD16* expression is significantly enhanced by salt in the roots of *arf7-1 arf19-1 ARF7pro:ARF7-GR* seedlings even without ARF7 activity under mock treatment in the presence of CHX alone (Fig. 2E). These data support our hypothesis that additional player(s) regulate *LBD16* expression in response to a prolonged salt stress, besides ARF7 and ARF19.

### Identification of upstream regulators of *LBD16* acting in response to salt

To expand our understanding of the molecular mechanism underlying the salt-induced LR plasticity phenotype of *lbd16* mutants and the observed salt-induced, auxin-independent regulation of *LBD16* expression, we set out to identify additional upstream regulator(s) of *LBD16* in response to salt stress. To this end, we performed Y1H screening by using a 1,309-bp promoter region upstream of the *LBD16* start codon as a bait and a collection of 1,956 Arabidopsis transcription factor genes as prey to find putative upstream regulators of *LBD16* (Pruneda-Paz et al. 2014). This identified more than 300 transcription factors that can bind to the promoter region of *LBD16*, including many basic helix–loop–helix (bHLH), APETALA2 (AP2)–ethylene-responsive element binding protein (EREBP), C2H2, and MYB-type transcription factors (Supplementary Data Set 1 and Fig. S5A).

**Figure 3.**
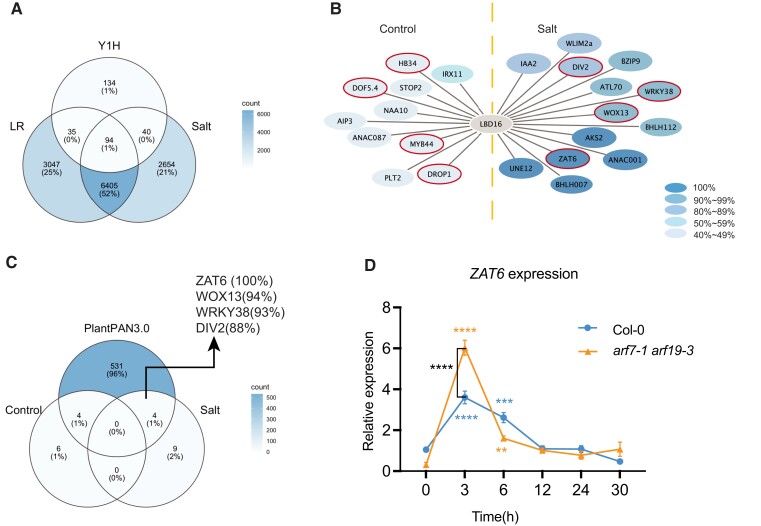
Identification of upstream regulators of *LBD16* in response to salt. **A)** Upstream regulators for *LBD16* mediating LR development in response to salt stress were selected by a comparative analysis among salt-induced root cell–type-specific transcriptomic response (Geng et al. 2013), LR development–associated genes (Voß et al. 2015), and putative *LBD16* upstream regulators via Y1H screening among 1,956 Arabidopsis transcription factor genes (Pruneda-Paz et al. 2014). **B)** Network inference prediction of *LBD16* upstream direct regulators by GRNBoost2 under control and salt conditions. The topology of the network shows genes that can directly regulate *LBD16*. Color-intensity indicates the robustness of the network prediction (40% to 100%). **C)** Comparative analysis of the *LBD16*-associated upstream network with *LBD16* promoter analysis on the basis of ChIP-seq evidence in PLANTPAN3.0 (Chow et al. 2019). **D)** Salt-induced expression pattern of *ZAT6* in roots of 7-d-old Col-0 and *arf7-1 arf19-3* seedlings. Data represent fold changes of ZAT6 expression normalized by the expression levels of the housekeeping gene At2g43770 and the expression of Col-0 at the 0-h time point (*n* = 4 to 5, Supplementary Data Set 5). Statistical analysis in **D)** was done using 2-way ANOVA followed by Tukey's multiple comparisons test. **P < 0.01, ***P < 0.001, and ****P < 0.0001. Putative LBD16 upstream regulators overlapped with PlantPAN3.0 analysis are highlighted with red-colored boders in **B)**.

To prioritize candidate transcription factors upstream of *LBD16* involved specifically in LR development in response to salt stress, we carried out a comparative analysis of public transcriptome data sets on root cell layers in response to salt stress (Geng et al. 2013) and gene expression during LR development (Voß et al. 2015). We established that 6,499 out of 9,193 salt-induced differentially expressed genes (DEGs) in Arabidopsis root cell layers overlap with genes induced during LR development (9,581 in total; Fig. 3A). We then compared these 6,499 DEGs to the 91 candidate upstream regulators of *LBD16* from our Y1H screening, ARF7 and ARF19, and LBD16 itself, resulting in a subset of 94 transcription factors that may be involved in LR development in response to salt stress (Fig. 3A; Supplementary Data Set 2).

We next performed a network inference analysis to construct an *LBD16*-associated network using GRNBoost2 (Moerman et al. 2019) based on the salt-induced time series coexpression data of these 94 transcription factor genes in the root stele cells from the salt response data set (Geng et al. 2013; Supplementary Data Set 3). To test the robustness of the network prediction, we computed the network inference 100 times and ranked all the predicted direct regulators of *LBD16* by how frequently they were predicted as an *LBD16* upstream direct regulator. We performed the same analysis using the expression data under control conditions (Supplementary Data Set 3). We thus identified 10 transcription factors that may regulate *LBD16* expression in control conditions and predicted that 13 act specifically in salt conditions (Fig. 3B). Among the potential salt-specific LBD16 upstream regulators, 5 were predicted with 100% frequency: ABA-RESPONSIVE KINASE SUBSTRATE 2 (AKS2, At1g05805), NAC DOMAIN CONTAINING PROTEIN 1 (ANAC001, At1g01010), ZAT6 (At5g04340), BASIC HELIX-LOOP-HELIX (BHLH007, At1g03040), and UNFERTILIZED EMBRYO SAC 12 (UNE12, At4g02590; Fig. 3B; Supplementary Data Set 3).

To narrow down the salt-induced candidates for further functional characterization, we performed an *LBD16* promoter analysis using the PlantPAN3.0 database to screen for upstream transcription factors that were shown to bind the *LBD16* promoter directly in existing chromatin immunoprecipitation sequencing (ChIP-seq) experiments (Chow et al. 2019). By comparative analysis of both the salt and control networks with the upstream regulator candidates from PlantPAN3.0 (Supplementary Data Set 3), we identified 4 candidate salt-specific transcription factors that can directly bind the *LBD16* promoter: ZAT6 (At5g04340), WUSCHEL RELATED HOMEOBOX 13 (WOX13, At4g35550), WRKY38 (At5g22570), and DIVARICATA2 (DIV2, At5g04760; Fig. 3C). Among these, only ZAT6 was predicted with 100% frequency in our network analysis (Fig. 3C; Supplementary Data Set 3). Further gene expression analysis showed that *ZAT6* expression peaks at 3 h into 125 mM NaCl treatment in both Col-0 and the *arf7-1 arf19-3* double mutant (Fig. 3D), suggesting that *ZAT6* expression after salt exposure may contribute to activate *LBD16* transcriptional activity independently of ARF7 and ARF19.

By analyzing *ZAT6* expression in the roots of the *arf7-1 arf19-1 ARF7pro:ARF7-GR* line under control and salt conditions, we confirmed that *ZAT6* expression does not rely on the induction of ARF7 in either control or salt conditions (Supplementary Fig. S5B). Additionally, by using a publicly available data set (Geng et al. 2013), we confirmed that *ZAT6* is expressed in the same cell layers as *LBD16* and that *ZAT6* and *LBD16* exhibit similar expression patterns in response to salt in the root stele—involved in LR development (Supplementary Fig. S5C), supporting a role for ZAT6 as a potential *LBD16* upstream regulator under salt. Collectively, our results reveal that ZAT6 is a potential upstream player regulating *LBD16* expression in response to salt in parallel to, but acting independently of, ARF7 or ARF19.

### ZAT6 acts as a positive upstream regulator of *LBD16* in response to salt treatment

**Figure 4.**
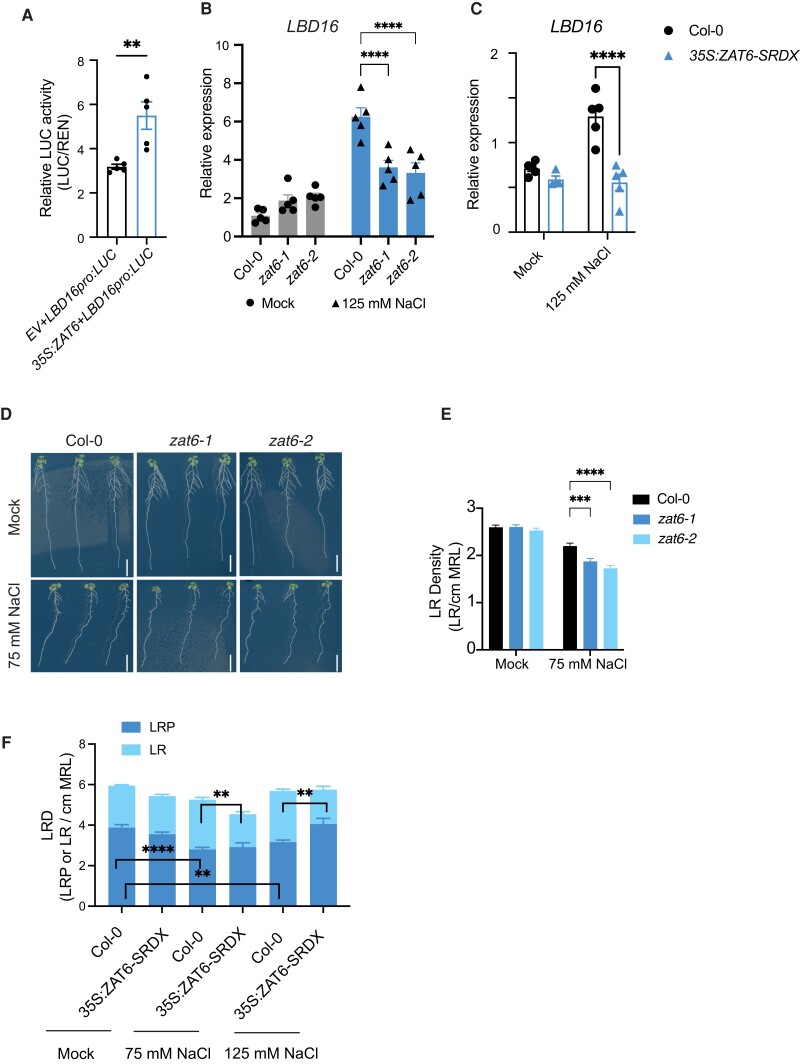
ZAT6 acts upstream of *LBD16* in response to salt. **A)** Overexpression of *ZAT6* (*35S:ZAT6*) enhances LUC activity derived from the *LBD16pro:LUC* reporter in *N. benthamiana* leaves (*n* = 5). EV, empty vector. **B)** Relative *LBD16* expression in response to mock (0 mM) or 125 mM NaCl treatment for 24 h in roots of Col-0, *zat6-1*, and *zat6-2* seedlings as determined by RT-qPCR. **C)** Relative *LBD16* expression in the roots of 7-d-old seedlings of a *35S:ZAT6-SRDX* line after 24 h mock treatment (0 mM NaCl) or salt (125 mM NaCl; *n* = 4 to 5). **D)** Representative photographs of roots from 10-d-old seedlings of Col-0, *zat6-1*, and *zat6-2* under mock (0 mM) and 75 mM NaCl treatments. Scale bars, 1 cm. **E)** Emerged LR density of 10-d-old seedlings of Col-0, *zat6-1*, and *zat6-2* under mock (0 mM NaCl) or 75 mM NaCl treatment. Data were collected from 3 independent experiments (*n* = total of 60 to 75 roots). **F)** Density of emerged LRs and nonemerged LRP in 10-d-old seedlings of Col-0 and the *35S:ZAT6-SRDX* line under mock (0 mM NaCl), 75 mM NaCl, or 125 mM NaCl treatment (*n* = 10 to 11). Data represent results of 2 independent experiments (for each *n* = 10 to 13). Data in **A to C)**, **E)**, and **F)** represent means ± SEM. Expression data in **B)** and **C)** were obtained from 4 biological replicates (approximately 40 to 45 roots were pooled as 1 replicate) and presented as the relative expression to mock condition after normalization by the reference gene At2g43770. Root RNA samples were obtained from 7-d-old seedlings transferred to half-strength MS agar plates alone or containing 125 mM NaCl for 24 h. MLR, main root length. Statistical analysis in **A)** was done using a *t* test. Statistical analyses in **B)** and **E)** were done using 2-way ANOVA followed by Dunnett's multiple comparisons test. Statistical analyses in **C)** and **F)** were done using 2-way ANOVA followed by Tukey's multiple comparisons test. **P < 0.01, ***P < 0.001, and ****P < 0.0001.

To understand the transcriptional regulation of *LBD16* by ZAT6, we validated the interaction of ZAT6 with the *LBD16* promoter using dual-luciferase assays in *Nicotiana benthamiana* leaves. Accordingly, we cloned the 1,309-bp region of the *LBD16* promoter upstream of the firefly *LUCIFERASE* (*LUC*) reporter gene to generate the reporter construct *LBD16pro:LUC*. The *ZAT6* coding region driven by the cauliflower mosaic virus (CaMV) 35S promoter was used as an effector construct. We coinfiltrated the reporter and effector constructs together with the internal control *Renilla* luciferase (REN) driven by the 35S promoter into *N. benthamiana* leaves using the *Agrobacterium* AgL0 strain. Notably, coinfiltration of *35S:ZAT6* with *LBD16pro:LUC* resulted in a higher relative luciferase activity compared to an empty 35S vector control (Fig. 4A), whereas ZAT10, which is closely related to ZAT6, did not enhance *LBD16* promoter activity (Xie et al. 2019; Supplementary Fig. S6A).

To understand whether ZAT6 acts upstream of *LBD16* under salt stress in planta, we assessed the expression of *LBD16* in roots of the loss-of-function mutants *zat6-1* (SALK_061991C) and *zat6-2* (SALK_050196) as well as a constitutive repression line of *ZAT6* by using a SUPERMAN REPRESSIVE DOMAIN X (SRDX) fusion, namely *35S:ZAT6-SRDX*. In both *zat6* mutant alleles (Supplementary Fig. S6B), we detected a significant decrease in *LBD16* expression at 24 h of 125 mM NaCl treatment, while *LBD16* expression in control conditions was not affected (Fig. 4B). Similarly, *LBD16* expression in the *ZAT6* repressor line *35S:ZAT6-SRDX* line was significantly lower compared to Col-0 at 24 h of salt treatment (Fig. 4C). Together, these results identify ZAT6 as a positive upstream regulator of *LBD16*, which is required for the salt-induced increase in *LBD16* expression in roots.

To further investigate whether ZAT6 is involved in LR formation under salinity, we transferred 4-d-old Col-0, *zat6-1*, and *zat6-2* seedlings to agar plates alone or containing 75 mM NaCl for 6 d and scored their RSA traits (Fig. 4, D and E). We observed no difference in main root length or average LR length under either control or salt stress conditions between Col-0 and *zat6* seedlings (Fig. 4D; Supplementary Fig. S6, C and D). Yet, we observed a lower density of emerged LRs in the *zat6-1* and *zat6-2* mutants compared to Col-0 under 75 mM NaCl treatment (Fig. 4, D and E). The drop in LR density was due to fewer emerged LRs under 75 mM NaCl (Supplementary Fig. S6E), as the main root length did not change significantly (Supplementary Fig. S6C).

We further evaluated LR development by scoring emerged LRs and nonemerged LRP in *35S:ZAT6-SRDX* under both control and salt conditions. We observed that the density of emerged LRs in the *35S:ZAT6-SRDX* line significantly decreases compared to that in Col-0 under 75 and 125 mM NaCl treatments whereas there was no difference in main root length between these 2 genotypes (Fig. 4F; Supplementary Fig. S7), consistent with the significantly lower *LBD16* expression in the *35S:ZAT6-SRDX* line compared to Col-0 in response to salt stress (Fig. 4C). Similar to the *lbd16-1* mutant (Supplementary Fig. S2C), LRP density did not decrease in the *35S:ZAT6-SRDX* line in response to salt stress relative to control conditions, while salt suppressed the formation of LRs in Col-0 (Fig. 4F). Collectively, the data suggest that ZAT6 is required for LRP development and LR emergence under salinity stress conditions.

### The ZAT6*–LBD16* pathway regulates cell wall remodeling in response to salt

**Figure 5.**
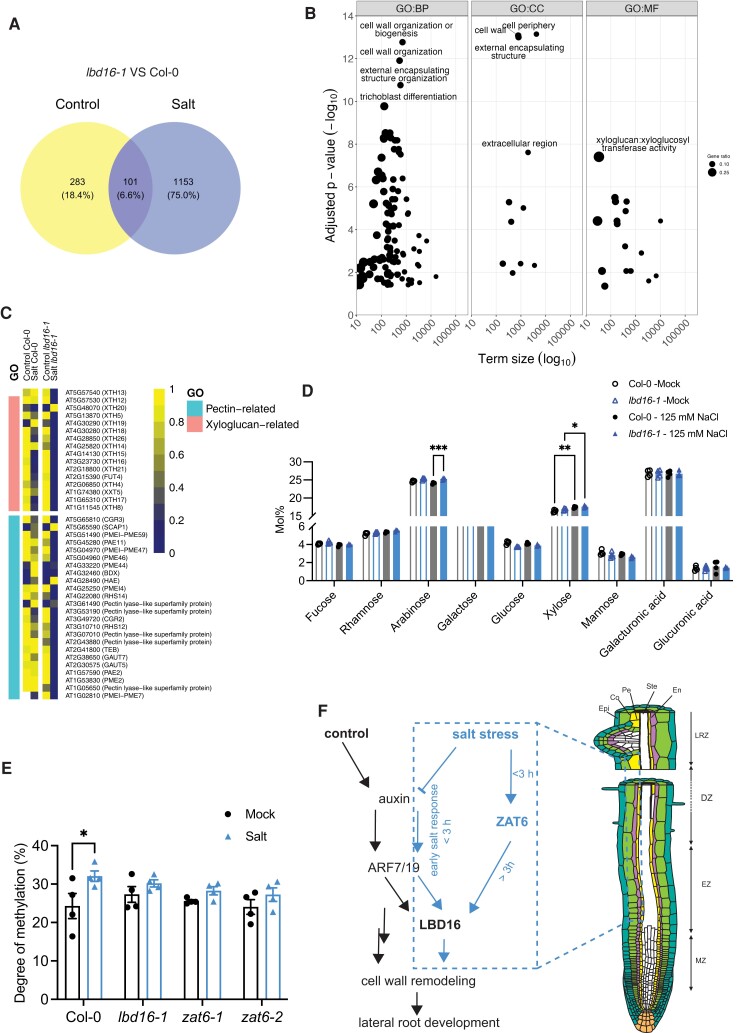
The *ZAT6–LBD16* pathway regulates cell wall remodeling in response to salt. **A)** Venn diagram showing the number of DEGs between 8-d-old *lbd16-1* and Col-0 after mock treatment (0 mM NaCl) or treatment with 130 mM NaCl for 6 h (no cutoff for log_2_[fold change], *P* < 0.05). **B)** GO term enrichment analysis of the 1,153 salt-induced genes in **A)**. BP, biological process; CC, cellular compartment; MF, molecular function. The *y* axis indicates the significance of the GO terms. The *x* axis indicates term size. **C)** Heatmaps showing the relative expression of genes listed in pectin- and xyloglucan-related GO terms in the roots of Col-0 and *lbd16-1* seedlings in control and salt conditions. **D)** Analysis of cell wall monosaccharide contents in the roots of 7-d-old *lbd16-1* and Col-0 after transfer to agar plates containing no (0 mM NaCl) or 125 mM NaCl for 24 h. Data are means ± SEM. Data were obtained from 4 independent biological replicates containing 60 to 80 roots each. Statistical analysis was done using 2-way ANOVA, followed by Tukey's multiple comparisons test. *P < 0.05, **P < 0.01, and ***P < 0.001. **E)** Degree of pectin methylation in Col-0, *lbd16-1*, *zat6-1*, and *zat6-2* under mock (0 mM NaCl) or 125 mM NaCl conditions. Data were collected from the roots of 7-d-old seedlings after transfer to mock (0 mM) or 125 mM NaCl for 72 h (*n* = 4 pools of 60 to 80 roots each). Data are means ± SEM. Statistical analysis was done using 2-way ANOVA followed by Bonferroni’s multiple comparisons test. *P < 0.05. **F)** Diagram of the LBD16-mediated molecular network in the main zones guiding LR development under control and salt stress conditions. The dashed box highlights the molecular pathways induced by high salt conditions identified by this study. Under control conditions, auxin and its downstream response factor ARF7 are involved in cell wall remodeling during LR development (Kumpf et al. 2013), in which the role of LBD16 was unclear. During the early salt stress (<3 h) response, root auxin signaling is inhibited while auxin signaling is still contributing to the regulation of *LBD16*. Salt activates *ZAT6* expression, whose encoding protein regulates *LBD16* during the long-term (>3 h) salt stress response to mediate downstream cell wall remodeling and subsequently LR development. Various root cell layers are represented by Ste (stele), Pe (pericycle), En (endodermis), Co (cortex), and Epi (epidermis), respectively. MZ, EZ, DZ, and LRZ denote meristematic zone, elongation zone, differentiation zone, and lateral root zone (root zone with emerged LRs), respectively. **F)** was adapted from https://doi.org/10.6084/m9.figshare.5143987.v4.

To gain insight into the molecular mechanisms of *LBD16-*mediated LR developmental plasticity in response to salinity, we analyzed the transcriptomes of roots from 8-d-old Col-0 and *lbd16-1* seedlings treated with 130 mM NaCl for 6 h by transcriptome deep sequencing (RNA-seq). Quality control using principal component analysis (PCA) and hierarchical clustering methods presented distinct expression patterns between salt and control conditions (Supplementary Fig. S8, A to C). In total, 384 genes were differentially expressed (false discovery rate [FDR] ≤ 0.05) between *lbd16-1* and Col-0 in control conditions, while we identified 1,254 significant DEGs under salt treatment. Among these 1,254 genes, 101 genes overlapped with the DEGs in control condition and 1,153 DEGs were specific to salt stress (Fig. 5A; Supplementary Data Set 4). To identify key molecular processes involved in the salt-induced *LBD16*-mediated root branching plasticity, we performed a gene ontology (GO) analysis using the salt-specific DEGs (Supplementary Data Set 4). Strikingly, some of the most significantly overrepresented GO terms were linked to cell wall organization (biological process [BP]), cell wall (cellular compartment [CC]), and xyloglucosyl transferase activity (molecular function [MF]; Fig. 5B; Supplementary Fig. S8D and Data Set 4).

Further investigation into the expression profiles of the genes enriched in these cell wall–related GO terms suggested that salt treatment more strongly downregulates the expression of pectin- and xyloglucan-related genes in the *lbd16-1* mutant compared to Col-0, while we also observed a subtle difference in their expression profiles between *lbd16-1* and Col-0 in control conditions (Fig. 5C). Among them, for example, *PME2*, *GALACTURONOSYLTRANSFERASE 7* (*GAUT7*), and *XTH19* were previously shown to be expressed in developing LRs (Atmodjo et al. 2011; Wachsman et al. 2020). In agreement, salt stress significantly enhanced the expression of *PME2* and *XTH19* in Col-0, whereas this induction was abolished in the *35S:ZAT6-SRDX* line likely due to the repressed activity of *LBD16* by ZAT6 (Fig. 4C; Supplementary Fig. S9). Together, our data suggest that the ZAT6–*LBD16* module is involved in salt-induced cell wall remodeling in roots.

To assess how the salt-induced ZAT6*–LBD16* module affects downstream cell wall modification, we surveyed changes in cell wall monosaccharide composition in the roots of Col-0, *lbd16-1*, *zat6-1*, and *zat6-2* in control conditions and at 24 h of 125 mM NaCl treatment by high-performance anion-exchange chromatography with pulsed amperometric detection (HPAEC-PAD) of isolated alcohol-insoluble residue (AIR) cell wall material. Salt exposure resulted in differences between Col-0 and *lbd16-1* in the relative amounts of arabinose and galactose, while the relative xylose amount increased in both Col-0 and *lbd16-1* after salt treatment (Fig. 5D). Although the overall salt-induced cell wall monosaccharide composition in *zat6-1* and *zat6-2* relative to Col-0 was similar to that of the *lbd16-1* mutant, we did not observe significant changes in the cell wall matrix composition of the *zat6* mutants at 24 or 72 h into salt treatment (Supplementary Fig. S10).

Since we detected salt-induced changes in *PME* and *PMEI* gene expression in the *lbd16-1* mutant relative to Col-0 (Fig. 5C), which were reported to be highly relevant for LR development (Wachsman et al. 2020), we further assessed the degree of pectin methylation in the roots of Col-0, *lbd16-1*, *zat6-1*, and *zat6-2* seedlings. We detected a significantly higher degree of pectin methylation in Col-0 at 72 h of salt treatment, while the changes in *lbd16-1*, *zat6-1*, and *zat6-2* mutants were much less noticeable (Fig. 5E). This finding is in agreement with the observation that salt enhances *PME2* expression in Col-0 but not in the *35S:ZAT6-SRDX* line, suggesting that *PME2* expression is dependent on ZAT6 activity and that salt-induced regulation of pectin methylation requires LBD16 and ZAT6 (Supplementary Fig. S9). In summary, these data indicate that salt activates the ZAT6*–LBD16* module to regulate downstream cell wall modification, positively contributing to LR developmental plasticity.

## Discussion

LRs play important roles in the acquisition of water and nutrients. Flexibility in when and where to branch is essential for a plant to reach an optimal RSA in a dynamic soil environment. We report here that LBD16 plays a major role in mediating root branching plasticity in response to salinity. We identified ZAT6 as a positive regulator that functions independently of auxin-induced ARF7 and ARF19 signaling to induce *LBD16* expression in long-term salt-stressed roots. Salt attenuates root auxin signaling during the early stress response, whereas it enhances the ZAT6–*LBD16* module to facilitate downstream cell wall remodeling, likely to promote LR development in response to salt, bypassing the canonical root branching pathways that function in control conditions (Fig. 5F).

Auxin plays an essential role during LR formation, and LBD16 acts as a transcription factor downstream of auxin, required for LR initiation occurring in the early differentiation zone of the Arabidopsis root (Goh et al. 2012; Du and Scheres 2018). Auxin signaling was also reported to be required for the induction of cell wall–related genes and for accommodation of the swollen pericycle cells in the endodermis during LR formation (Kumpf et al. 2013; Vermeer et al. 2014). We show that during early salt stress (3 h into salt treatment), the auxin–ARF7/ARF19 module may still mediate *LBD16* expression (Fig. 2E). Using the auxin reporter line C3PO, we show that salt suppresses auxin output (DR5v2 signals) in the main root zones where LRs are primed (oscillation zone) and initiate (differentiation zone), which may contribute to the decreased density of nonemerged LRP in Col-0 (Supplementary Fig. S2C). However, salt-enhanced *LBD16* promoter activity and *LBD16* expression in the main root zones, including LR zone, differentiation zone, and the elongation zone (Fig. 1D; Supplementary Fig. S3, B and C), suggest the existence of an auxin-independent LBD16-mediated pathway acting during salt stress from LR priming to LR emergence. Similarly, in the LR domains, we observed a diminished auxin input signal (mDII/DII) and constant DR5v2 signals in the early stage LRP (Stage I through Stage IV) at 6 h into salt treatment (Supplementary Fig. S4, A and B).

Although salt had an overall negative effect on root auxin levels and signaling (Fig. 2, A to C), we found auxin-responsive genes such as *PIN-FORMED 3* (*PIN3*, At1g70940), *LIKE AUXIN RESISTANT 1* (*LAX1*, At5g01240), and several *IAA*s (*IAA9*, *IAA11*, and *IAA13*) in the upregulated GO term “response to hormone” in our RNA-seq analysis of *lbd16-1* compared to Col-0 specifically at 6 h of salt treatment, but not in control conditions (Supplementary Data Set 4), indicating that there might be feedback regulation of auxin signaling in the *lbd16-1* mutant upon salt treatment. Interestingly, LBD18 reported to be functionally redundant with LBD16 under control conditions (Lee et al. 2009) interacts with ARF7 via the Phox and Bem1 (PB1) domain of ARF and binds to the *ARF19* promoter to positively regulate its expression (Pandey et al. 2018). Therefore, it is still plausible that the redundant LOB transcription factors and their dimerization also play a role during rooting in salt (Lee et al. 2009; Goh et al. 2012; Lee et al. 2017). Moreover, SUMOylation of ARF7 to block its activation of *LBD16* (Orosa-Puente et al. 2018) and salt-activated auxin downstream TARGET OF RAPAMYCIN (TOR) signaling to promote the translation of ARF7, ARF19, and LBD16 (Jamsheer et al. 2022; Stitz et al. 2023) were previously shown to contribute to LR development. Although whether these abovementioned auxin downstream transcriptional and post-transcriptional mechanisms contribute to salt-induced root branching phenotype of *lbd16* mutants is still unknown, our results do provide evidence for a ZAT6-dependent transcriptional pathway that acts independently of auxin, regulating cell wall remodeling during LR development under salt stress. Notably, changes in cell wall components were shown to directly affect auxin response during plant development (Aryal et al. 2020; Jewaria et al. 2021); therefore, it is plausible that the lower auxin accumulation and signaling in the main root and LR domains in response to salt is partially contributed by ZAT6–*LBD16*-mediated cell wall remodeling occurring during LR development. Together, it appears that salt activates positively acting pathway(s) in which LBD16 is required to mediate cell wall remodeling and promote LRP development under high-salinity conditions. In the *lbd16* mutants, this positive contribution is lacking, resulting in the observed lower root branching phenotype.

Our network inference analysis indicates that, besides ARF7 and ARF19, various transcription factors, such as ZAT6 (encoded by At5g04340), WRKY38 (encoded by At5g22570), WOX13 (encoded by At4g35550), and DIV2 (encoded by At5g04760) are potential *LBD16* upstream regulators (Fig. 3). Among them, we show that ZAT6 might be involved in regulating LR density independently of ARF7 and ARF19 (Fig. 3D), by binding to the *LBD16* promoter and upregulating *LBD16* expression in response to salt stress (Fig. 4). Although it is still unknown how ZAT6 is activated by salt in plant roots, *ZAT6* was previously found to be expressed in the pericycle cells and to contribute to limiting Na^+^ accumulation in the shoots during salt stress (Fabado et al. 2022). ZAT6 was also reported to be phosphorylated directly by MPK6 and implicated in regulation of seed germination of Arabidopsis in saline conditions (Liu et al. 2013). Salt may induce MPK6 activity within 15 min to activate downstream salt-responsive gene expression in Arabidopsis seedlings (Gigli-Bisceglia et al. 2022). In addition, MPK3/MPK6 signaling is also involved in LR development via regulating downstream cell wall remodeling pathways (Huang et al. 2019; Zhu et al. 2019). It is thus possible that salt might activate ZAT6 activity to promote LR formation via the MPK6-mediated signaling pathway. However, the other top candidates predicted by the network inference analysis also have the potential to regulate root branching in response to salt. For instance, WOX13 (encoded by At4g35550) was previously identified in a data set regarding LR initiation (Vanneste et al. 2005). DIVARICATA 2 (DIV2) (encoded by At5g04760) is a MYB protein that has been shown to play a negative role during salt stress and is required for ABA signaling (Song et al. 2016; Fang et al. 2018).

We did not detect significant changes in the composition of cell wall monosaccharides in the *zat6* mutants in response to salt stress compared to Col-0 (Supplementary Fig. S10), which may be explained by the fact that ZAT6 acts upstream to activate *LBD16* activity under high-salt conditions to subsequently modulate the expression of cell wall–related genes. Nevertheless, salt significantly upregulated the expression of cell wall remodeling genes such as *PME2* and *XTH19* in Col-0, but not in the *35S:ZAT6-SRDX* repressor line where both ZAT6 and LBD16 activities are suppressed (Fig. 4D; Supplementary Fig. S9), suggesting that the ZAT6–*LBD16* module plays a central role in regulating cell wall remodeling genes under high-salt conditions. Together with the similar expression pattern of *ZAT6* and *LBD16* in root cell layers upon salt treatment and the similar degree of pectin methylation in *zat6* and *lbd16-1* mutants (Supplementary Fig. S5C; Fig. 5E), our data support a role for ZAT6 in cell wall remodeling, especially by affecting pectin methylation via LBD16. It is likely that the misregulation of cell wall–related genes by salt application and consequent cell wall changes in the *zat6* and *lbd16-1* mutants compared to Col-0 may disturb cell wall loosening during LR emergence, resulting in the lower LR density seen in *zat6* and *lbd16* mutants compared to Col-0. To further understand how the cell wall undergoes remodeling during rooting in high salinity, investigations on whether the LR phenotype and cell wall changes in the *lbd16-1* and *zat6* mutants are salt specific are still needed.

In summary, our data show that salt inhibits root auxin levels and signaling and in parallel it activates alternative pathway(s) that help sustain root branching under salt stress. We identified such a pathway—a salt-activated *ZAT6*–*LBD16* module–promoting root branching in response to salt via altering cell wall remodeling. This study provides a theoretical framework for developmental plasticity under stress; we propose that the identified ZAT6–*LBD16* module allows plants to mitigate the effects of inhibition of the core developmental root branching program by salt, allowing optimization of root architecture in stressful conditions.

## Materials and methods

### Plant materials, growth conditions, and stress treatments

All Arabidopsis (*A. thaliana*) wild-type, mutant, and transgenic lines used in this work are in the Col-0 accession, except for the auxin reporter line C3PO (in Col-Utrecht; Küpers et al. 2023). The T-DNA knockout insertion lines *lbd16-1* (SALK_095791) and *lbd16-2* (SALK_040739) were obtained from Prof. Malcolm Bennett (University of Nottingham) and the Nottingham Arabidopsis Stock Centre (NASC, http://arabidopsis.info/), respectively. Both *lbd1*6 alleles were reported previously (Orosa-Puente et al. 2018) and were genotyped again prior to being used in our study. The T-DNA knockout mutants *zat6-1* (SALK_061991C) and *zat6-2* (SALK_050196) were obtained from the NASC and genotyped for confirmation. The genomic complementation line *LBD16genomic:GFP* in *lbd16-1* (gLBD16) and the *ARF7pro:ARF7-GR* line were obtained from previously published reports (Okushima et al. 2007; Goh et al. 2012). Primers used for genotyping are listed in Supplementary Table S1.

For seedling pregrowth in petri plates for all root phenotyping experiments, Arabidopsis seeds were surface sterilized in 70% (v/v) ethanol for 1 min and then immersed in 30% (v/v) bleach with Triton X-100 (10 *µ*L per 50 mL) for 10 min, followed by washing with sterilized Milli-Q water at least 6 times. Disinfected seeds were sown on half-strength MS medium (including B5 vitamins, Duchefa Biochemie B.V.) containing 0.1% (w/v) MES (Duchefa Biochemie B.V.) with pH 5.8 and 0.8% (w/v) plant agar (Lab M, MC029) and then stratified at 4 °C in the dark for 2 d followed by pregrowth in a growth chamber at 22 °C under a 16-h light/8-h dark photoperiod and 65% to 70% humidity.

For LR phenotypic characterization, 4-d-old seedlings were transferred to fresh agar plates containing half-strength MS (including B5 vitamins, Duchefa Biochemie B.V.) supplemented with 0 mM, 75 mM NaCl, or 125 mM NaCl and allowed to grow for 6 d.

For analyses of *LBD16genomic:GFP* expression patterns and GUS activity derived from the *LBD16pro:GUS* transgene, 6-d-old seedlings were transferred into liquid half-strength MS medium (including B5 vitamins, Duchefa Biochemie B.V.) containing 0.1% (w/v) MES (Duchefa; pH 5.8) alone or with 125 mM NaCl on a shaker for 6 h to avoid hypoxia during the treatments before microscopy analysis.

For time course expression studies of *LBD16* in adult Col-0 plants grown in liquid half-strength MS (including B5 vitamins, Duchefa Biochemie B.V.) hydroponically, plants were grown under a 12-h light/12-h dark photoperiod at 20 °C for 4 wk before being treated with 125 mM NaCl for 3, 6, 12, 18, or 24 h. For time course expression of *LBD16* and *ZAT6* in Col-0 and *arf7-1 arf19-3*, seedlings were grown on half-strength MS (including B5 vitamins, Duchefa Biochemie B.V.) agar plates containing 0.1% (w/v) MES (pH 5.8) and 1% (w/v) Dashin agar (Duchefa Biochemie B.V.) under a 16-h light/8-h dark photoperiod at 22 °C for 7 d, and then they were treated with 125 mM NaCl for 3, 6, 12, 18, 24, or 30 h.

For the expression of *ZAT6* in *zat6-1* and *zat6-2* T-DNA mutants, seedlings were grown on half-strength MS (including B5 vitamins, Duchefa Biochemie B.V.) agar plates containing 0.1% (w/v) MES (pH 5.8) and 1% (w/v) Dashin agar under a 16-h light/8-h dark photoperiod at 22 °C for 10 d before roots were harvested for gene expression analysis.

For RNA-seq analysis, seeds were sown and pregrown on 11 × 11 cm nylon mesh. Eight-day-old seedlings were transferred to half-strength (including B5 vitamins, Duchefa Biochemie B.V.) MS agar plates containing 0.1% (w/v) MES (pH 5.8) and 1% (w/v) Dashin agar (Duchefa Biochemie B.V.) alone or with 130 mM NaCl for 6 h before roots were harvested for total RNA isolation.

For cell wall analysis, 7-d-old seedlings of Col-0, *lbd16-1*, *zat6-1*, and *zat6-2* grown on 11 cm × 1 cm nylon mesh strips were transferred to half-strength MS (including B5 vitamins, Duchefa Biochemie B.V.) agar plates containing 0.1% (w/v) MES (pH 5.8) and 0.8% (w/v) plant agar (Lab M, MC029) alone or with 125 mM NaCl for 24 h or 3 d (72 h) before roots were harvested for analysis.

### Cloning

For the Y1H assay, a 1,309-bp *LBD16* promoter sequence upstream of the start codon was cloned into the gateway vector pDONR207, which was then Sanger sequenced before the cassette containing the *LBD16* promoter was recombined into a modified gateway-compatible destination bait vector pAbAi (Danisman et al. 2012) containing the *AbAr* gene (*AUR-1C*) reporter through LR reaction, and then restriction enzyme digestions were performed to assess the final plasmid.

For *LBD16pro:GUS* analysis, the *LBD16* promoter sequence was first cloned into pDONR207, then recombined into the pFAST-G04 binary vector for plant transformation and expression.

For the *35S:ZAT6-SRDX* repressor line, the full-length *ZAT6* coding sequence (with the stop codon removed) was first cloned into pDONR221, then subcloned into pGWB119 vector for plant transformation.

For luciferase assays, pDONR221 carrying the 1,309-bp *LBD16* promoter sequence was used for subcloning into pGWB435 carrying a *LUC* reporter, and pDONR221 carrying the *ZAT6* and *ZAT10* coding sequences was used for subcloning into the pH7WG2 binary vector for overexpression.

All constructs for plant transformation were transformed into *Agrobacterium* (*Agrobacterium tumefaciens*) strain AgL0 by electroporation and then used for transformation of Col-0 plants by floral dipping (Zhang et al. 2006).

### RSA assay

Four-day-old seedlings of Col-0, *lbd16-1*, the *gLBD16* (in *lbd16-1*) complementation line, *zat6-1*, and *zat6-2* mutants were transferred to half-strength MS medium alone or containing 75 or 125 mM NaCl in square petri plates, which were then placed at a 70° angle on racks and scanned on Day 6 (10-d-old seedlings) after transfer. Main root and LR phenotypes were traced by ImageJ with the SmartRoot plugin (Lobet et al. 2011). Data were extracted as CSV files and then processed with R.

### LR primordium assay

Four-day-old seedlings of Col-0, *lbd16-1*, and *35S:ZAT6-SRDX* lines were transferred to half-strength MS agar plates alone or containing 75 or 125 mM NaCl. Petri plates containing the transferred seedlings were placed at a 70° angle on racks at 21 °C under a 16-h light/8-h dark photoperiod and 65% to 70% humidity. For counting LRP and emerged LR under a microscope, seedlings were fixed 6 d after transfer by immersion first in 20% (v/v) methanol with 4% (v/v) hydrochloric acid at 57 °C for 20 min prior to immersion in 7% (w/v) NaOH in 60% (v/v) ethanol at room temperature for 15 min, followed by rehydration in 40%, 20%, and 10% (all v/v) ethanol for 5 min each. The fixed seedlings were stored in 10% (v/v) ethanol at 4 °C prior to microscopy analysis. For microscopy analysis, a Leica DM5200 microscope with Nomarski optics was used, and the roots were immersed in 50% (v/v) glycerol on microscopy slides for scoring LRP. The LRP stages were determined according to a previous report by Malamy and Benfey (1997).

### Y1H

The final *pLBD16pro:AbAi* plasmid was transformed into the yeast (*Saccharomyces cerevisiae*) strain pJ69-4α as bait. A yeast library containing the coding sequences of 1,956 Arabidopsis transcription factor genes (Pruneda-Paz et al. 2014) cloned into the pDEST-22 vector was transformed into the strain pJ69-4A as prey clones as previously described (de Folter and Immink 2011). Positive yeast colonies were selected and confirmed by using different synthetic defined (SD, Takara) media prepared according to the manufacturer's instructions. Prior to screening, the strain carrying the *pLBD16pro:AbAi* bait construct was tested for autoactivation by using an empty strain carrying the empty prey vector (pDEST-22) under various concentrations of aureobasidin A (AbA, Takara; 0, 50, 100, 150, 200, and 400 ng/mL); 200 ng/mL AbA was selected for library screening. For mating-based screening, all the colonies carrying individual prey clones were grown overnight in SD−Trp medium in 96-well V bottom plates in a 28 °C shaker. The yeast strain carrying the *pLBD16pro:AbAi* construct was grown in a 28 °C shaker overnight in SD−Ura medium. Mating of yeast strains was carried out by spotting 5 *µ*L of 10-fold diluted overnight culture of both bait and prey strains in 1 spot on agar plate containing complete SD medium without glucose at 28 °C for 2 to 3 d. The diploid yeast colonies containing both *pLBD16pro:AbAi* and prey clones were resuspended into 100 *µ*L sterilized Milli-Q water, and 5 *µ*L was spread onto SD−Trp−Ura medium containing 200 ng/mL AbA. Positive interactions were observed after 2 to 3 d of growth at 28 °C.

### Histochemical GUS staining

Six-day-old seedlings of independent *LBD16pro:GUS* homozygous transgenic lines in the Col-0 background were transferred to liquid half-strength MS medium alone or containing 125 mM NaCl and placed on a shaker for 6 h. Then seedlings were transferred into GUS staining buffer containing 50 mM sodium phosphate buffer (pH 7.2), 10 mM EDTA, 0.1% (v/v) Triton X-100, 2.5 mM each of potassium ferricyanide and potassium ferrocyanide, and 1 mg/mL 5-bromo 4-chloro-3-indolyl-b-glucuronic acid (Duchefa) and vacuum infiltrated for 15 min at room temperature before incubation at 37 °C for 1.5 h. Samples were cleared in clearing solution containing water (30 mL): chloral hydrate (80 g):glycerol (10 mL) and mounted on microscope slides for imaging. A Leica DM5200 microscope with Nomarski optics was used for imaging with a 20× dry objective.

### Confocal microscopy and auxin reporter analysis

For the detection of GFP in *LBD16genomic:GFP* (in the *lbd16-1* background), 6-d-old seedlings were transferred to liquid half-strength MS medium alone or containing 125 mM NaCl on a shaker for 6 h to avoid hypoxia. After treatment, roots were fixed using 4% (w/v) paraformaldehyde (PFA) in 1× phosphate-buffered saline (PBS) for 1 h at room temperature with gentle agitation. After fixation, the roots were washed twice for 1 min each time in 1× PBS. The roots were then transferred and immersed in ClearSee solution containing 10% (w/v) xylitol, 15% (w/v) sodium deoxycholate, and 25% (w/v) urea at room temperature with gentle agitation overnight. Seedling cell membranes were counterstained for 30 min in a 0.1% (w/v) calcofluor white (in ClearSee solution). Cleared and stained roots were mounted onto slides with ClearSee solution for confocal imaging.

For quantification of LBD16genomic:GFP fluorescence in root zones, the average fluorescent intensity was measured in manually drawn regions of interest over the GFP channel from different root zones. Measurements were taken in the root center (*z*-position). The average fluorescent background signal was also measured in different zones in each image. Subsequently, this value was used to correct the mean fluorescent intensity.

For auxin response analysis during salt stress, 6-d-old Arabidopsis Col-0 seedlings carrying the triple color auxin reporter C3PO (Küpers et al. 2023) were transferred to liquid half-strength MS medium alone or containing 125 mM NaCl and placed on a shaker for 6 h. After treatment, roots were fixed in 4% (w/v) PFA and cleared overnight using ClearSee solution. Roots were stained in 0.1% calcofluor white (in ClearSee solution) for 1 h for cell membrane staining. Cleared roots were mounted onto slides in ClearSee solution. The roots were visualized using a Leica TCS SP5II confocal laser scanning microscope with a 40× NA = 1.20 water-immersion objective. mTurqoise2 and calcofluor white were excited using a 405-nm diode laser; mVenus and GFP were excited using a 488-nm argon-ion laser, and tdTomato was excited using a 552-nm laser. Detection was configured as follows: calcofluor white was detected at 410 to 430 nm; mTurqoise2 was detected at 468 to 495 nm; GFP was detected at 500 to 550 nm; Venus was detected at 524 to 540 nm; tdTomato was detected at 571 to 630 nm. *Z*-stacks were acquired in 2.0-*µ*m intervals, with the pinhole set to 2.0 Airy units. Virtual ratio images between channels were generated using the FIJI plugin Calculator Plus (Fiji). Ratios between pixel signal intensities of nuclei were calculated on SUM-slice projection of DII (C1; Venus) and mDII (C2; tdTomato) after subtracting the background signal (value = 4). Nuclei in each LRP area were manually selected as “region of interest” (ROI, Fiji) for quantifying average auxin input through ratio image (max C2/max C1/ROI) and average auxin output per ROI (max C3/ROI).

### Luciferase assay

A Dual-Luciferase Reporter (DLR) system (Promega) was employed to test the activation of *LBD16pro:LUC* by *35S:ZAT6*. *Agrobacterium* cultures carrying *LBD16pro:LUC*, the P19 silencing suppressor gene, *35S:ZAT6*, *35S:ZAT10*, or an empty *35S:pH7WG2* vector constructs were mixed in a 1:1:3 ratio (v/v/v) with OD_600_ = 0.5 for each construct and coinfiltrated into the leaves of 4-wk-old *N. benthamiana* plants. *35S:REN* (with OD_600_ = 0.2) was used as an internal control for infiltration efficiency, with a final 3:1:1:0.5 ratio (v/v/v/v) for effector, promoter, P19, and *35S:Renilla*. Three days after infiltration, leaf samples of equal size were collected for protein extraction using a Dual-Luciferase Reporter Assay System kit (Promega, Fitchburg, USA). A Glomax 96 microplate luminometer (Promega, Fitchburg, USA) was used to measure luciferase activities. The LUC/REN values were calculated to obtain relative LUC activity.

### Reanalysis of published transcriptome data

For the analysis of public microarray data sets of LR development (Voß et al. 2015) and root response to salt stress (Geng et al. 2013), data analysis was performed using R with R packages provided by Bioconductor (Gentleman et al. 2004). The R packages affy and simpleaffy were employed for reading .CEL files of the corresponding data sets, quality control, background adjustment, and normalization (Gautier et al. 2004; Wilson and Miller 2005). Quality control was performed by checking whether the scale factors of different chips were within 3-fold of one another, and 3′/5′ ratios of each sample were below 3 using simpleaffy (Wilson and Miller 2005). GCRMA was used to perform global normalization (Irizarry et al. 2006). PCA was applied to check if the overall variability of the samples reflected their grouping. Based on the affy_ATH1_array_elements-2010-12-20 table in TAIR, probe sets that were annotated to hybridize to multiple loci in the Arabidopsis genome were removed from further analysis. DEGs were identified using the LIMMA package in R (Ritchie et al. 2015). The Benjamini–Hochberg method was used to correct for multiple testing. Genes with at least 2-fold change of expression (log_2_ fold change ≥1) and *P* ≤ 0.001 in data set (Geng et al. 2013) or *P* ≤ 0.05 in data set (Voß et al. 2015) were considered as significantly DEGs.

### Network inference

Network inference was performed using GRNBoost2 (Moerman et al. 2019). The overlapping DEGs from public transcriptomic data sets, potential upstream regulators from Y1H screening, and published known upstream regulator(s) were together used as input gene list for the network inference (Okushima et al. 2007). Expression data of these genes for the network inference were extracted from previously published public data sets (Geng et al. 2013) to predict the upstream *LBD16* regulators under both control and salinity conditions. Expression data from 2 time points for control treatment and 6 time points for salinity conditions were used for network prediction. To enhance the robustness of the network analysis, the algorithm was applied 100 times for expression data under different conditions (control and salinity conditions). For each run, predicted direct regulators of *LBD16* were extracted from all inferred interactions using a custom Python script. The frequency of each predicted *LBD16*-related interaction was calculated over these 100 predictions. To prioritize the list of potential *LBD16* regulators, only interactions with a frequency equal to or higher than 80% were listed for further analysis for the salinity conditions, and interactions with frequency ≥40% were selected for control conditions to deal with higher variability in the network predictions (Supplementary Data Set 3).

### RNA-seq analysis

Eight-day-old seedlings grown a thin layer of nylon mesh on half-strength MS agar plates were transferred to fresh agar plates containing either 0 or130 mM NaCl for 6 h. Roots were dissected for harvest and stored in liquid nitrogen until total RNA isolation. Approximately 40 roots were pooled as 1 biological replicate. Total RNA was extracted using combined TRipure and RNA isolation kit (Qiagen). Three biological replicates were used for sequencing.

Total RNA was used for RNA library preparation suitable for Illumina HiSeq paired-end sequencing using an Illumina TruSeq stranded mRNA kit for polyA mRNA selection. Then mRNA was processed directly including RNA fragmentation, first- and second-strand cDNA synthesis, adapter ligation, and final library amplification according to the manufacturer's protocol. The final library was eluted in 30 *µ*L elution buffer followed by quality assessment using a Bioanalyzer 2100 DNA1000 chip (Agilent Technologies) and quantified on a Qubit platform (Life Technologies).

Prepared libraries were pooled and diluted to 6 pM each for TruSeq Paired End v4 DNA clustering on 1 single flow cell lane using a cBot device (Illumina). Final sequencing was carried out on an Illumina HiSeq 2500 instrument using 126, 7, 126 flow cycles for sequencing paired-end reads plus index reads. All steps for clustering and subsequent sequencing were done according to the manufacturer's protocol. Reads were demultiplexed by using CASAVA 1.8 software (Illumina Inc., San Diego, CA, USA). All sample preparations and sequencing were done by the Genomics lab of Wageningen University and Research, Business Unit Bioscience.

Read quality was assessed using FastQC (https://www.bioinformatics.babraham.ac.uk/projects/fastqc/), and low-quality reads were removed using “trim galore!” (https://www.bioinformatics.babraham.ac.uk/projects/trim_galore/). Reads were mapped to the Arabidopsis TAIR10 genome using Salmon (Patro et al. 2017).

Differential gene expression was analyzed using DESeq2 (Love et al. 2014) under different contrasts (*lbd16-1* vs Col-0 in both control and salinity conditions) using a FDR cutoff of 0.05. The counts of the top 5,000 DEGs were used for PCA. Expression heatmap of sample-to-sample distances was calculated from the log_2_-transformed count data for overall gene expression. Clustering of 12 RNA-seq samples was carried out to show the overview of relationships between genotypes and treatments. GO term enrichment analysis was performed for the NaCl-induced DEGs in *lbd16-1* vs Col-0 with the R package gProfiler (Raudvere et al. 2019).

### Gene expression analysis

For expression studies of *LBD16* in Col-0, *arf7-1 arf19-1 ARF7pro:ARF7-GR*, *zat6-1*, *zat6-2*, and *35S:ZAT6-SRDX*, 7-d-old seedlings pregrown on mesh strips on the surface of half-strength MS agar plates were transferred to fresh half-strength MS agar plates alone (0 mM) or containing 125 mM NaCl. The roots were collected after 3, 6, or 24 h treatment as specified in the figure legends for analysis.

For *LBD16* and *ZAT6* expression in *arf7-1 arf19-3*, 7-d-old seedlings grown on mesh strips on half-strength MS agar plates were transferred to fresh half-strength MS agar plates alone (0 mM) or containing 125 mM NaCl for 3, 6, 12, 24, or 30 h before roots were harvested for total RNA extraction.

For time course analysis of *LBD16* expression, Col-0 plants were hydroponically grown in liquid half-strength MS medium containing 0.1% (w/v) MES (pH 5.8) for 4 wk and then were transferred to fresh liquid half-strength MS medium alone (0 mM) or containing 125 mM NaCl for 3, 6, 12, 18, or 24 h before roots were harvested for total RNA isolation.

For confirmation of ZAT6 expression in *zat6-1* and *zat6-2* T-DNA mutants compared with Col-0, roots from 10-d-old seedlings were used for total RNA isolation. Total RNA was extracted with RNA isolation kits (NZYtech, Portugal). RNA quality and concentration were assessed prior to cDNA synthesis. An iScript cDNA synthesis kit (Bio-Rad) was used for first-strand cDNA synthesis. The primers used for qPCR are listed in Supplementary Table S1. 2x qPCRBIO SyGreen Mix Lo-ROX (PCRBIOSYSTEMS, UK) was used for qPCR analysis. qPCR analyses were performed using a CFX96 or CFX384 connect real-time PCR detection system (Bio-Rad).

### IAA measurements

Seven-day-old seedlings pregrown on a mesh strip on agar plates containing half-strength MS medium under a 16-h light/8-h dark photoperiod at 22 °C were transferred to fresh half-strength MS agar plates alone or containing 125 mM NaCl (Duchefa). After 1, 3, 6, or 12 h after transfer, the roots were dissected with a sharp blade, weighed, flash frozen in liquid nitrogen, and stored at −80 °C until analysis. For IAA extraction, approximately 60 roots (11.8 mg ± 3.0) were pooled as 1 biological replicate. Frozen material was ground to a fine powder and extracted with 1 mL of 10% (v/v) methanol containing 100 nM ^13^C-labeled ^13^C_6_-IAA as internal standard (CIL, Cambridge Isotope Laboratories, Inc.). The extraction was carried out according to a previous report with minor modifications (Floková et al. 2014). Namely, a Strata-X 30 mg/3 mL SPE column (Phenomenex) was used.

For detection and quantification of IAA by LC-MS/MS, sample residues were dissolved in 100 *µ*L acetonitrile:water (20:80, v/v) and filtered using a 0.2-*µ*m nylon centrifuge spin filter (BGB Analytik). IAA was identified and quantified by comparing retention time and mass transitions with IAA standard using a Waters Xevo TQ-S mass spectrometer equipped with an electrospray ionization source coupled to an Acquity UPLC system (Waters) as previously described (Guhl et al. 2021). Chromatographic separations were conducted using acetonitrile:water (containing 0.1% [v/v] formic acid) on an Acquity UPLC BEH C18 column (2.1 mm × 100 mm, 1.7 *µ*m, Waters) at 40 °C with a flow rate of 0.25 mL/min. The column was equilibrated for 30 min with acetonitrile:water (20:80, v/v) containing 0.1% (v/v) formic acid. Samples were analyzed by injecting 5 *µ*L, followed by elution using program of 17 min in which the acetonitrile fraction linearly increased from 20% (v/v) to 70% (v/v). The column was washed after every sample by increasing the acetonitrile fraction to 100% over 1 min and maintaining this concentration for 1 min. The acetonitrile fraction was decreased to 20% (v/v) over 1 min and maintained at this concentration for 1 min before injecting the next sample. The capillary voltage was set to 3.5 kV, the source temperature and the desolvation temperature to 150 and 350 °C, respectively. Multiple reaction monitoring (MRM) mode was used for identification and quantification by comparing retention times and MRM transitions (+176.25 > 103.2; +176.25 > 130.2) with the IAA standard. MRM transitions and cone voltages (30 V) were set using the IntelliStart MS Console. Data acquisition and analysis were carried out using MassLynx 4.1 (TargetLynx) software (Waters) and were further processed in Microsoft Excel. The final IAA content in each sample was normalized by the corresponding internal standard recovery and sample weight. All values were normalized to the 1-h control time point.

### Cell wall analysis

Seven-day-old seedlings of Col-0, *zat6-1*, *zat6-2*, and *lbd16-1* pregrown on mesh strips on the surface of half-strength MS agar plates under a 16-h light/8-h dark photoperiod at 22 °C were transferred to fresh half-strength MS agar plates alone or containing 125 mM NaCl. Roots were harvested into 2-mL tubes after 24 or 72 h of treatment and ground to a fine powder. One milliliter of prewarmed 70% (v/v) ethanol was added to the tubes, followed by vortexing. This solution was spun at maximum speed 21,130 × *g* at room temperature for 30 s and the supernatant discarded, after which this ethanol washing step was repeated and the supernatant was discarded again. A mixture of 50% (v/v) chloroform and 50% (v/v) methanol was added to the pellet, and the tube was mixed by gently inverting the tube at least 5 times. Tubes were spun at maximum speed 21,130 × *g* at room temperature for 30 s, and the supernatant was discarded. Acetone was added to the tubes, the tubes spun at max speed 21,130 × *g* at room temperature for 30 s, and the supernatant was discarded. This acetone wash was repeated 3 times. Tubes were left to dry with open lids overnight in a fume hood. The resulting AIR was used for analysis.

For analysis of cell wall monosaccharide composition, 1 to 2 mg AIR was weighed in 2-mL screw cap tubes and used for extraction of neutral cell wall sugars and uronic acids as described (Yeats et al. 2016). HPAEC-PAD was performed on a biocompatible Knauer Azura HPLC system, equipped with an Antec Decade Elite SenCell detector. Monosaccharides were separated on a Thermo Fisher Dionex CarboPac PA20 column BioLC guard (3 × 30 mm) and analytical (3 × 150 mm) column as previously described (Bacete et al. 2022). Briefly, samples were diluted with ultrapure water, ribose was added as an internal standard, and separation was performed with a solvent gradient of (A) water, (B) 10 mM NaOH, and (C) 700 mM NaOH at 0.4 mL/min flow rate. The program was as follows (all steps in v/v): 0 to 25 min: 20% B; 25 to 28 min: 20% to 0% B, 0% to 70% C; 28 to 33 min: 70% C; 33 to 35 min: 70% to 100% C; 35 to 38 min: 100% C; 38 to 42 min: 0% to 20% B, 100% to 0% C; 42 to 60 min: 20% B.

The degree of pectin methylesterification was analyzed as described (Lionetti et al. 2007) with minor modifications. One milligram of AIR was saponified in 0.2 mL 250 mM NaOH for 1 h at room temperature and neutralized with 1 M HCl. After centrifugation for 2 min at 16,000 × *g*, the supernatant was diluted 1:10 with water to 50 *µ*L and was incubated with an equal volume of 0.1 M sodium phosphate buffer (pH 7.5) containing 0.03 units alcohol oxidase (Sigma A2404) for 15 min at room temperature with shaking. After addition of 100 *µ*L 0.02 M 2,4-pentanedione in 2 M ammonium acetate and 0.5 M acetic acid, samples were incubated at 68 °C for 10 min, briefly cooled on ice, and then transferred to 96-well microtiter plates. Absorbance was measured at 412 nm in a Tecan infinite 200Pro microplate reader and calibrated against a 0 to 350 *µ*M formaldehyde standard curve. The degree of pectin methylesterification was calculated as the molar ratio of methanol to uronic acid determined via HPAEC-PAD analysis.

### Statistical analysis and R scripts

Individual data points for Figs. 2, C and D, and 3D are shown in Supplementary Data Set 5. GraphPad Prism software and R were used for statistical analyses (Supplementary Data Set 6). For the proportion of nonemerged LRP and emerged LRs, a generalized linear model was fitted (glm(cbind(`non-emerged`,emerged) ∼ genotype*condition, family = binomial(link = “logit”)), and statistical differences were derived from least squares mean analysis with custom contrast (emmeans R package 1.8.1-1).

Customized scripts used in this study were deposited in a Github repository (https://github.com/liyiyunnn/LBD16_in_salt).

### Accession numbers

Accession numbers: *LBD16* (At2g42430); *ZAT6* (At5g04340); *ZAT10* (AT1G27730); *AKS2* (At1g05805), *ANAC001* (At1g01010), *BHLH007* (At1g03040), and *UNE12* (At4g02590); and *WRKY38* (At5g22570), *WOX13* (At4g35550), and *DIV2* (At5g04760). Raw RNA-seq data were deposited in EBI under the accession number E-MTAB-13345 (https://www.ebi.ac.uk/biostudies/arrayexpress/studies/E-MTAB-13345).

## Supplementary Material

koad317_Supplementary_Data

## Supplementary data

The following materials are available in the online version of this article.

**Supplementary Figure S1.** Root phenotypic analysis of the *lbd16-2* mutant and Col-0 under control and salt conditions (supports Fig. 1).

**Supplementary Figure S2.** Root phenotypic analysis of Col-0, *lbd16-1*, and the *LBD16* complementation line (*lbd16-1 LBD16genomic-GFP*; supports Fig. 1).

**Supplementary Figure S3.** Salt-induced *LBD16* expression patterns and *LBD16pro:GUS-*derived GUS activity in the root of Col-0 seedlings (supports Fig. 1).

**Supplementary Figure S4.** Salt-affected auxin response in the main root and early stages of LRP of Arabidopsis wild-type plants and expression of *LBD16* in *arf7-1 arf19-1 ARFpro:ARF7-GR* (supports Fig. 2).

**Supplementary Figure S5.** Characterization of LBD16 upstream transcription factors by Y1H screening and expression patterns of *ZAT6* in *arf7-1 arf19-1 ARF7pro:ARF7-GR* and Col-0 (supports Fig. 3).

**Supplementary Figure S6.** Characterization of ZAT6 as an *LBD16* upstream regulator and root phenotype of its T-DNA knockout alleles (supports Fig. 4).

**Supplementary Figure S7.** Root phenotypic characterization of the *35S:ZAT6-SRDX* line (supports Fig. 4).

**Supplementary Figure S8.** Quality control of RNA-seq analysis of Col-0 and *lbd16-1* and identification of cell wall–related GO term (supports Fig. 5).

**Supplementary Figure S9.** Expression of *PME2* and *XTH19* in the roots of Col-0 and *35S:ZAT6-SRDX* (supports Fig. 5).

**Supplementary Figure S10.** Cell wall analysis of Col-0 and loss-of-function mutants of *LBD16* and *ZAT6* in response to salt stress (supports Fig. 5).

**Supplementary Table S1.** Primers used in this study.

**Supplementary Data Set 1.** Y1H identified potential upstream regulators of *LBD16*.

**Supplementary Data Set 2.** Reanalysis of public transcriptomic data sets.

**Supplementary Data Set 3.** Network inference analysis.

**Supplementary Data Set 4.** RNA-seq analysis of Col-0 and *lbd16-1* mutant in response to 6 h salt treatment.

**Supplementary Data Set 5.** Individual data points for Figs. 2, C and D, and 3D.

**Supplementary Data Set 6.** Results of statistical analysis.

## Dive Curated Terms

The following phenotypic, genotypic, and functional terms are of significance to the work described in this paper:

ARF19 Gramene: AT1G19220
ARF19 Araport: AT1G19220

CIL Gramene: AT4G25990
CIL Araport: AT4G25990

HSL2 Gramene: AT5G65710
HSL2 Araport: AT5G65710

ZAT6 Gramene: AT5G04340
ZAT6 Araport: AT5G04340

XTH19 Gramene: AT4G30290
XTH19 Araport: AT4G30290

LBD33 Gramene: AT5G06080
LBD33 Araport: AT5G06080

PIN3 Gramene: AT1G70940
PIN3 Araport: AT1G70940

SOS3 Gramene: AT5G24270
SOS3 Araport: AT5G24270

WRKY46 Gramene: At2g46400
WRKY46 Araport: At2g46400

XTH9 Gramene: AT4G03210
XTH9 Araport: AT4G03210

CYP79B2 Gramene: AT4G39950
CYP79B2 Araport: AT4G39950

IAA13 Gramene: Os03g0742900
IAA13 Araport: Os03g0742900

ARF7 Gramene: AT5G20730
ARF7 Araport: AT5G20730
