# Supplementary material for: Root branching under high salinity requires auxin-independent modulation of LATERAL ORGAN BOUNDARY DOMAIN 16 function
Source: Plant Cell. 2023 Dec 23;36(4):899–918. doi: 10.1093/plcell/koad317 (PMC10980347; doi:10.1093/plcell/koad317)
Supplement: koad317_Supplementary_Data [file koad317_supplementary_data.zip › Supplemental Data Zhang et al [74].docx]

**Supplemental Data**

**Supplemental Figure S1**. Root phenotypic analysis of the *lbd16-2* mutant and Col- 0 under control and salt conditions (Supports Figure 1).

**Supplemental Figure S2**. Root phenotypic analysis of Col-0*, lbd16-1* and the *LBD16* complementation line (*lbd16-1 LBD16genomic-GFP*) (Supports Figure 1).

**Supplemental Figure S3.** Salt-induced *LBD16* expression patterns and *LBD16pro:GUS-*derived GUS activity in the root of Col-0 seedlings (Support Figure 1).

**Supplemental Figure S4.** Salt-affected auxin response in the main root and early stages of lateral root primordia (LRP) of Arabidopsis wild-type plants and expression of *LBD16* in *arf7-1 arf19-1 ARFpro:ARF7-GR* (Supports Figure 2).

**Supplemental Figure S5.** Characterization of LBD16 upstream transcription factors by yeast one-hybrid screening and expression patterns of *ZAT6* in *arf7-1 arf19-1 ARF7pro:ARF7-GR* and Col-0 (Supports Figure 3).

**Supplemental Figure S6.** Characterization of ZAT6 as an *LBD16* upstream regulator and root phenotype of its T-DNA knockout alleles (Supports Figure 4).

**Supplemental Figure S7.** Root phenotypic characterization of the *35S:ZAT6-SRDX* line (Supports Figure 4).

**Supplemental Figure S8.** Quality control of RNA-seq analysis of Col-0 and *lbd16-1* and identification of cell wall–related GO term (Supports Figure 5).

**Supplemental Figure S9.** Expression of *PME2* and *XTH19* in the roots of Col-0 and *35S:ZAT6-SRDX* (Support Figure 5).

**Supplemental Figure S10.** Cell wall analysis of Col-0 and loss-of-function mutants of *LBD16* and *ZAT6* in response to salt stress (Supports Figure 5).

**Supplemental Table S1.** Primers used in this study.


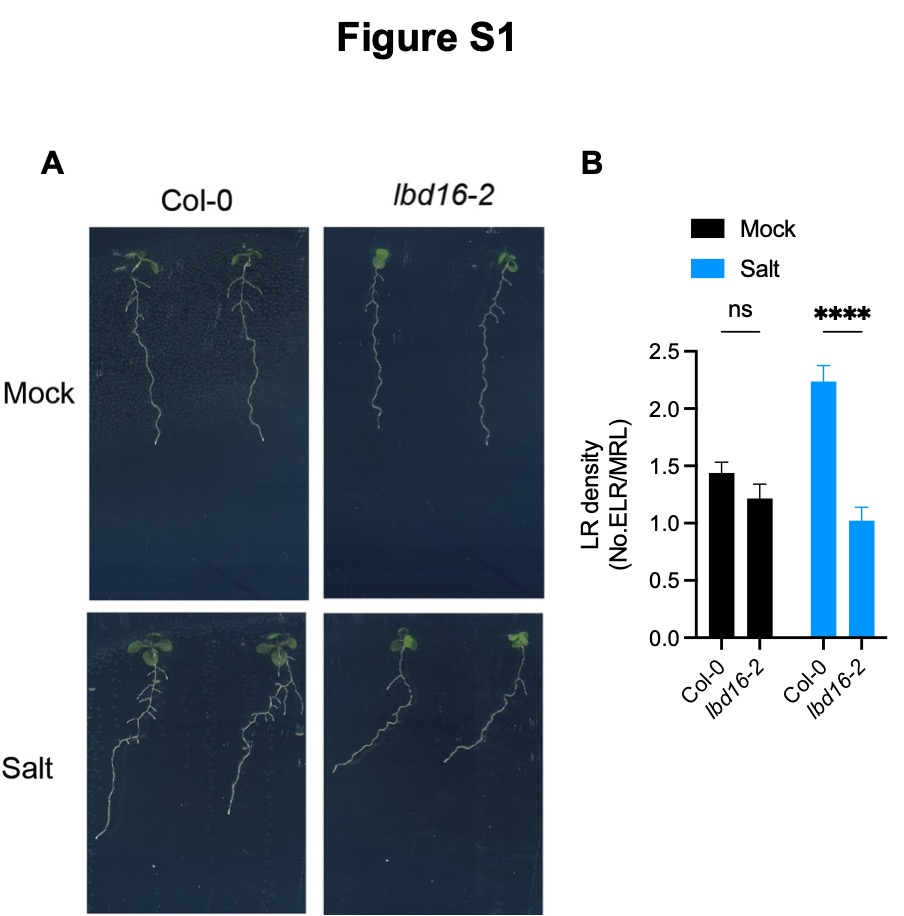


**Supplemental Figure S1. Root phenotypic analysis of the *lbd16-2* mutant and Col-0 under control and salt conditions (Supports Figure 1).**

**A)** Representative photographs of *lbd1- 2* and Col-0 seedlings grown under control (0 mM NaCl) or 75 mM NaCl. **B)** Emerged lateral root (LR) density in Col-0 and *lbd16-2* under control (6-day-old seedlings) and 75 mM NaCl conditions (10-day old seedlings) (n=15–20). Four-day-old seedlings were transferred to agar plates containing half-strength MS alone or containing 75 mM NaCl with addition of 1% (w/v) sucrose for 2 or 6 days before roots were scanned for emerged LR number quantification. MRL, main root length. Data in B represent means ± SEM. Statistical analysis in B was done using two-way ANOVA, followed by Tukey’s multiple comparisons test. **** *P*<0.0001.

**Supplemental Figure S2. Root phenotypic analysis of Col-0*, lbd16-1* and the *LBD16* complementation line (*lbd16-1 LBD16genomic-GFP*) (Supports Figure 1).**

**A)** Main root length of 10-day-old Col-0*, lbd16-1* and *LBD16* genomic complementation line (*lbd16-1 LBD16genomic-GFP*) (n=15–20). Data are representative of at least 3 independent experiments. **B)** Density of emerged lateral roots (LRs) in Col-0, *lbd16-1* and *lbd6-1* *gLBD16* complementation line in control (0 mM) and salt conditions (75 mM or 125 mM NaCl) (n= 15–20). **C)** Density of non-emerged lateral root primordia (LRP) and LRs in *lbd16-1* and Col-0 under control and salt conditions (n= a total of 25–34, a pool of 3 independent experiments). Four-day-old seedlings were transferred to agar plates containing half-strength MS alone or containing 75 mM or 125 mM NaCl for 6 days (10-day old seedlings) before roots were scanned for root clearing and quantification of LRP and LR under a microscope. Data in **A-C** represent means ± SEM. MRL in **B** and **C** represents main root length. Statistical analyses in **A-C** were done using two-way ANOVA followed by Tukey’s multiple comparisons test. * *P* < 0.05, ** 0.05<*P*<0.01, *** *P*<0.001, **** *P*<0.0001.

**Supplemental Figure S3. Salt-induced *LBD16* expression patterns and *LBD16pro:GUS-*derived GUS activity in the root of Col-0 seedlings (Support Figure 1).**

**A)** Salt-induced *LBD16* expression patterns in roots of 4-week-old hydroponically grown Col-0 plants (n=4–5). Relative expression was normalized by both the housekeeping gene At2g43770 and expression at the 0-h time point. Statistical analysis was done using a t-test with Welch’s correction. * *P* < 0.05, *** *P*<0.001. **B)** *LBD16* promoter activity as indicated by GUS staining in different main root zones of 6-day-old seedlings after treatment with 0 mM or 125 mM NaCl. **C)** GUS activity staining derived from the *LBD16pro:GUS* reporter in developing LRP under control and 125 mM NaCl conditions. Six-day old seedings were pre-grown on agar plates containing half-strength MS medium, and treated with liquid half-strength MS medium alone or containing 125 mM NaCl with gentle shaking for 6 h prior to GUS staining. Scale bars, 0.1 mm in **B** and **C**. DZ, differentiation zone; LRZ, lateral root zone (root zone with emerged LR). Images in B and C represent data from at least two independent transgenic lines from 3 independent experiments.

**Supplemental Figure S4. Salt-affected auxin response in the main root and early stages of lateral root primordia (LRP) of Arabidopsis wild-type plants and expression of *LBD16* in *arf7-1 arf19-1 ARFpro:ARF7-GR* (Supports Figure 2).**

**A)** Auxin input signal as indicated by the ratio of mDII/DII in developing LRP (stage I through IV) of 6-day old wild-type Col-Utrecht seedlings in control and salt (125 mM NaCl) conditions for 6 h (n=3–8). **B)** Auxin output DR5v2 activity in early stages of LRP of 6-day old wild-type Col-Utrecht seedlings in control and salt (125 mM NaCl) conditions for 6 h (n=3–11). **C)** Relative *LBD16* expression in the *arf7-1 arf19-1 ARF7pro:ARF7-GR* seedlings were treated with 1 μM IAA, 2 μM dexamethasone (DEX) and/or 10 μM cycloheximide (CHX) in control and salt conditions after 3 h with 125 mM NaCl treatment in comparison to mock treatment under 0 mM NaCl (n=4–5). Lowercase letters (a to e) above the bars indicate the statistical differences, and data with the same letter are not significantly different. Expression values were normalized by the housekeeping gene At2g43770. Data in **A**–**C** represent means ± SEM. Statistical analyses in **A** and **B** were done using a t-test. Statistical analysis in **C** was done using two-way ANOVA followed by Bonferroni's or Tukey’s multiple comparisons test. ***P*<0.01, *****P* < 0.0001.

**Supplemental Figure S5. Characterization of LBD16 upstream transcription factors by yeast one-hybrid screening and expression patterns of *ZAT6* in *arf7-1 arf19-1 ARF7pro:ARF7-GR* and Col-0 (Supports Figure 3).**

**A)** Distribution of transcription factor families as putative upstream regulators of LBD16 via yeast one-hybrid (Y1H) screening. **B)** Expression of *ZAT6* in *arf7-1 arf19-1 ARF7pro:ARF7-GR* seedlings treated with 1 μM IAA, 2 μM dexamethasone (DEX) and/or 10 μM cycloheximide (CHX) after 3 h or 6 h of treatment with 125 mM NaCl in comparison to mock treatment (n=4). Lowercase letters (a to d) above the bars indicate the statistical differences, and data with the same letter are not significantly different. **C)** Relative *LBD16* and *ZAT6* expression in different root cell types of Col-0 in control and salt conditions from Geng et al., 2013. MS represents control treatment without NaCl at 0 h. Expression values in **B** were normalized by the housekeeping gene At2g43770 and mock treatment under 0 mM NaCl. Data in **B** represent means ± SEM. Statistical analysis in **B)** was done using two-way ANOVA followed by Tukey’s multiple comparisons test.

**Supplemental Figure S6. Characterization of ZAT6 as an *LBD16* upstream regulator and root phenotype of its T-DNA knockout alleles (Supports Figure 4).**

**A)** LUC activity driven by *LBD16* promoter in *Nicotiana benthamiana* leaves after co-infiltration with *35S:ZAT6* or *35S:ZAT10* (n=4–5). **B)** RT-qPCR analysis of *ZAT6* expression in the T-DNA knockout *zat6-1* and *zat6-2* lines (n=5–6). The expression is shown as fold-change normalized by a housekeeping gene in comparison with expression in Col-0. **C)** Main root length of 10-day old Col-0, *zat6-1* and *zat6-2* seedlings. **D)** Average lateral root length (aLRL) in 10-day old Col-0, *zat6-1* and *zat6-2* seedlings after transfer to fresh medium lacking salt (0 mM) or containing 75 mM for 6 days. **E)** Number of emerged lateral roots (LRs) in 10-day old Col-0, *zat6-1* and *zat6-2* seedlings after transfer to half-strength MS agar plates lacking salt (0 mM) or containing 75 mM NaCl for 6 days. Data in **A-E)** represent means ± SEM. Data in C-E were collected from 3 independent experiments (n= a total of 60–75 roots). Statistical analysis in **A)** was done using one-way ANOVA. Statistical analysis in **B)** was done using a t-test with Welch’s correction, and in **C-E)** were done using two-way ANOVA followed by Tukey’s multiple comparisons test. * *P*<0.05, ***0.001<*P*<0.01, **** *P* < 0.001.


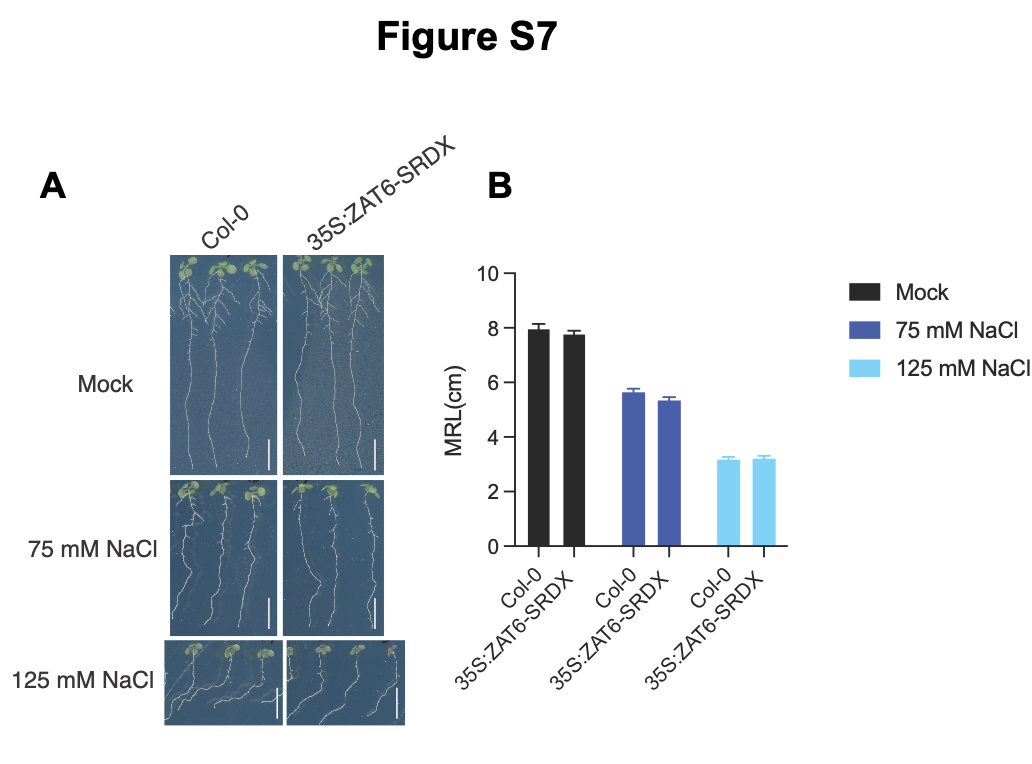


**Supplemental Figure S7. Root phenotypic characterization of the *35S:ZAT6-SRDX* line (Supports Figure 4).**

**A)** Representative photographs of 10-day old Col-0 and *35S:ZAT6-SRDX* seedlings under mock, 75 mM NaCl and 125 mM NaCl conditions. Scale bars, 1cm. **B)** Main root length of 10-day old Col-0 and *35S:ZAT6-SRDX* seedlings. Data in **B** represent means ± SEM and are representative of two independent experiments (n=10–15 in each experiment). Statistical analysis in **B** was done using two-way ANOVA followed by Šídák's multiple comparisons test.

**Supplemental Figure S8. Quality control of RNA-seq analysis of Col-0 and *lbd16-1* and identification of cell wall–related GO term (Supports Figure 5).**

**A)** Principal component analysis of the top 5000 differentially expressed genes in the RNA-seq analysis. Each dot represents a sample. Color coded according to the treatments, and shape of dot coded according to the time points. **B)** Clustering of the samples in the RNA-seq analysis. The Euclidean distance of the log2-transformed counts is shown in the heatmap. A darker color in the heatmap means the corresponding samples are more correlated. **C)** Dispersion plot of the RNA-seq analysis processed by DEseq2. The dispersion decreases smoothly for genes with higher expression and eventually reaches an asymptote, which can be considered as the biological variability that is present in the dataset. **D)** Expression profiles of genes from other cell wall–related GO term (besides pectin and xyloglucan-related GO terms shown in Figure 5 in Col-0 and *lbd16-1* under control and salt conditions.

**Supplemental Figure S9. Expression of *PME2* and *XTH19* in the roots of Col-0 and *35S:ZAT6-SRDX* (Support Figure 5).**

The expression is shown as fold-change normalized by the housekeeping gene At2g43770, compared with Col-0 values under mock conditions (n=4–5 pools of 40–45 roots). Roots of 7-day-old Col-0 and *35S:ZAT6-SRDX* seedlings were transferred to either fresh half-strength MS agar plates or containing 125 mM NaCl for 24 h before they were harvested for gene expression analysis. Data represent means ± SEM. Statistical analyses were done using two-way ANOVA followed by Tukey’s multiple comparisons test. * *P* <0.05, ***P*<0.01, *** *P*<0.001, **** *P*<0.0001.

**Supplemental Figure S10. Cell wall analysis of Col-0 and loss-of-function mutants of *LBD16* and *ZAT6* in response to salt stress (Supports Figure 5).**

**A)** Cell wall monosaccharides composition of the roots from 7-day old Col-0, *lbd16-1*, *zat6-1* and *zat6-2* seedlings after 24 h treatment with 125 mM NaCl or mock-treated (n=3–4). **B)** Cell wall monosaccharide composition of the roots from 7-day old Col-0, *lbd16-1*, *zat6-1* and *zat6-2* seedlings after 72 h treatment with 125 mM NaCl or mock treatment (n=3-4). Data in A and B represent means ± SEM and were obtained from 3–4 pools of samples containing 60–80 roots. Statistical analyses in **A** and **B** were done using two-way ANOVA followed by Tukey’s multiple comparisons test. * *P* <0.05, ***P*<0.01, *** *P*<0.001, **** *P*<0.0001.

**Supplemental Table S1.** Primers used in this study.

| **Primer name** | **Sequence (5’ to 3’)** | **Purpose** |
| --- | --- | --- |
| Geno_*lbd16-1*_F | TTTCTTCCTTTTTGCTTTGCC | SALK_095791 genotyping gene-specific forward primer |
| Geno_*lbd16-1*_R | CAATGGCCAGTGACTTAAAGC | SALK_095791 genotyping gene-specific reverse primer |
| Geno_*lbd16-2*_F | CGCCAAAATCTTGAGTAAACG | SALK_040739 genotyping gene-specific forward primer |
| Geno_*lbd16-2*_R | TCATTTCTGTTTCAATTCTCCG | SALK_040739 genotyping gene-specific reverse primer |
| Geno_*zat6-1*_F | AGTAAGCGAAAAGCTTTTCCG | SALK_061991C genotyping gene-specific forward primer |
| Geno_*zat6-1*_R | GGGGCACTATAGTGGCACTAG | SALK_061991C genotyping gene-specific reverse primer |
| Geno_*zat6-2*_F | AGACGAAGAAGAAGGCAGGTC | SALK_050196 genotyping gene-specific forward primer |
| Geno_*zat6-2*_R | TCGTACTTTGGCGAAACATTC | SALK_050196 genotyping gene-specific reverse primer |
| Left border primer SALK T-DNA genotyping | ATTTTGCCGATTTCGGAAC | ALK left border primer for genotyping |
| qP_LBD16_F | TCATCATCAAACCGGAGGAG | AT2G42430 qPCR forward primer |
| qP_LBD16_R | GCCTGAAGCTCACCTAAATCG | AT2G42430 qPCR reverse primer |
| qP_ZAT6_F | AGTGTGAAGTCGCACGTTTG | AT5G04340 qPCR forward primer |
| qP_ZAT6_R | TGCTGACGTGGCTTGTAGAC | AT5G04340 qPCR reverse primer |
| qP_PME2_F | GACGGAAGCGGTGACTTTAC | AT1G53830 qPCR forward primer |
| qP_PME2_R | ATAGTTTTGCCACGGCCATC | AT1G53830 qPCR reverse primer |
| qP_XTH19_F | CTTGTAGCCCAATGCTCTGC | AT4G30290 qPCR forward primer |
| qP_XTH19_R | GAAACTTGTCCCTGGTAACTCTG | AT4G30290 qPCR reverse primer |
| at2g43770 | TATCATTGGATCTTGCAGTAGTG | Housekeeping gene, Dekkers et al., 2012 |
| at2g43770 | ACATCGTCGATTCTAAAGACTTC | Housekeeping gene, Dekkers et al., 2012 |
