# Supplementary material for: Root branching under high salinity requires auxin-independent modulation of LATERAL ORGAN BOUNDARY DOMAIN 16 function
Source: Plant Cell. 2023 Dec 23;36(4):899–918. doi: 10.1093/plcell/koad317 (PMC10980347; doi:10.1093/plcell/koad317)
Supplement: koad317_Supplementary_Data [file tpc.00421.2023-s10.pdf › tpc.00421.2023-s10.pdf]

THE PLANT CELL

Root branching in salt requires auxin-independent modulation of LBD16 function

Yanxia Zhang, Yiyun Li, Thijs De Zeeuw, Kilian Duijts, Dorota Kawa, Jasper Lamers, Kristina Munzert, Hong-Fei Li, Yutao Zou, A Meyer, Jinxuan Yan, francel verstappen, Yixuan Wang, Tom Gijsberts, Jielin Wang, Nora Gigli-Bisceglia, Timo Engelsdorf, Aalt van Dijk, and Christa Testerink

Review Timeline:

Submission Date:	16-May-2023
Editorial Decision:	22-Jun-2023
Revision Received:	13-Sep-2023
Editorial Decision:	07-Oct-2023
Revision Received:	17-Nov-2023
Accepted:	18-Nov-2023

Prof. Dr. Christa Testerink
Wageningen University
Wageningen, 6708 PB
Netherlands

Dear Dr. Testerink:

We have received reviews of your manuscript entitled "Root branching in salt requires auxin-independent modulation of LBD16 function." Thank you for submitting your best work to The Plant Cell. The editorial board agrees that the work you describe is substantive, falls within the scope of the journal, and may become acceptable for publication, pending revision and potential re-review.

The reviewers have an overall positive view of this manuscript. However, they do still raise concerns to be addressed. Most of the comments can be addressed by revisions to the text or revisions of the figures. Additional data should be added to confirm that the different plant ages used for different experiments, noted by reviewer 2, is not having a major effect on the results. Also, the authors should better support their conclusion about LBD16 expression being increased in arf7/19 (2D); this was also raised by reviewer #2. Providing some data which tissue is the ZAT16 mediated regulation of LBD16 is taking place would add to the manuscript.

Please contact us if there are ambiguous comments or if you wish to discuss the revision.

Given the nature of the comments, we are offering you 60 days from when we have issued this decision to complete the revision. If a revision is not returned within this time frame, and if you have not been granted an extension, we will withdraw the manuscript, which will leave you free to submit the work elsewhere. If you need an extension, we encourage you to contact us at any point before the 60 days have passed.

When you are ready to submit the revised version, please upload a highlighted copy that indicates all changes made in response to the editor and reviewer recommendations. Include an itemized list of all changes made in response to each of the reviewer's suggestions in the "Response to Reviewers" section; please note that reviewers do not have access to your cover letter, nor was this decision letter shared with them.

Thank you for the privilege of reviewing your work. We look forward to receiving your revised manuscript.

Sincerely,

The Plant Cell Editorial Board

Please note the following:

-The Plant Cell now requires authors to complete and submit an author revisions checklist upon submission of a revised manuscript. The aim of the checklist is to aid authors in preparing a high-quality manuscript, facilitate the review and assessment of revised manuscripts, and help to ensure that journal standards are maintained across the board. If your manuscript is accepted, the completed checklist will be published as supplemental material attached to the article online. Please download a copy of the checklist (pdf fillable form) at this link, for submission with your revised manuscript: https://tpc.msubmit.net/html/Author_Revisions_Checklist.pdf.

-Supplemental materials should be restricted to large datasets and tables, presentation of replicates, and validation of reagents, methods, or genotypes. Any data that are used to support the major claims must be in the main manuscript. Supplemental figure legends must indicate what figure in the main manuscript is supported by the supplemental data presented. Please justify how each of the supplemental figures meet the criteria.

-Sampling methods and nature of "biological replicates" should be described precisely (i.e. different plants, parts of plants, pooled tissue, independent pools of tissue, sampled at different times, etc), along with a clear description of and rationale for any statistical analyses conducted. The reader should know exactly what was sampled; what forms the basis of the calculation of any means and statistical parameters reported. This is also necessary to ensure that proper statistical analysis was conducted.

-Want to add this revision deadline to your calendar? Click below!

-- Reviewer comments:

Reviewer #1 (Comments for the Author):

Zhang et al. report on identifying ZAT16, a new upstream regulator of _LBD16_ expression mediating the induction of its expression in response to salt treatment in Arabidopsis roots. Zhang et al. further provide evidence that in response to salt treatment, this ZAT16-LBD16 module is required for the expression of cell wall modifying enzymes and thus suggest that modulation of root cell wall composition in response to salt treatment is an important element of the plant response to this stimulus.

The entry point of this manuscript is the observation that single _lbd16_ mutants present defects in LR emergence in response to salt treatment and that salt treatments lead to an increase of _LBD16_ expression independently of its already characterised upstream auxin-dependent regulators ARF7 & ARF19. To identify possible upstream regulators of _LBD16_ during salt treatment, the authors performed a Y1H screen followed by a systems analysis of public datasets. They narrowed it down to a short list of transcription factors, among them ZAT16. _ZAT16_ expression is salt-induced. Loss of function and repressor versions of ZAT16 fail to up-regulate LBD16 expression in response to salt properly. These mutants also phenocopy the _lbd16_ phenotype in response to salt. Finally, a transcriptomic analysis of _lbd16_ mutant in response to salt treatment uncovered an enrichment of genes linked to cell wall remodelling. The authors show that the composition of the cell wall is altered in response to salt treatment and this change in composition depends on the ZAT16-LBD16 module.

The manuscript is very high quality, and the findings are novel and interesting. The data support the conclusions. Yet, I have a few points to improve further the manuscript.

Major points:

**The main element missing in the manuscript is in which tissues is LBD16 acting in response to salt treatment. LBD16 is classically reported to be involved in the early phase of LR development (esp. Stage 1); here it appears it also plays a role in a later phase. The effects of _lbd16_ mutation are on emergence and salt triggers _LBD16_ expression in overlying tissues. I find these observations very interesting. Where is ZAT16 expressed? in overlying tissues?
Are the cell wall modifications reported related to changes in the primordium and/or the overlying tissues?
Corollary to this, where is _LBD16_ expressed in response to salt treatment in the in the _ZAT16_ mutants (loss of function or _ZAT16-SRDX_)?**

A second point is related to Fig 2D. The authors write "after 6 h salt treatment LBD16 expression was increased in the arf7-1;arf19-3 double mutant compared to Col-0". Yet, only a weak increase is visible on the graph and it does not appear to be different from the null hypothesis. The wording should be adapted. This key result is further confirmed with the ARF7-GR in arf7/19 background, so I do not doubt its validity, I am however surprised that the increase is much more marked (2E, Mock) than in arf7-1;arf19-3. Why that?

Minor points:

- l101: double "identified"
- l154: missing call to supp figure (Fig. 1c and Fig. Sc)

Reviewer #2 (Comments for the Author):

**In this paper, Zhang et al. investigated the role related to lateral root development of LBD16 in salinity conditions. LBD16 is a well-studied gene assigned a function in LR; it belongs to the LBD family of transcription factors. Doing a series of elegant experiments, they concluded that under salinity, LBD16 is regulated by an alternative non-canonical pathway. Auxin signaling is repressed under salinity conditions, and LBD16 is induced by ZAT6 (identified in this work as an upstream modulator), triggering cell wall remodeling and LR development.
The manuscript is, in general, well-written and illustrated with minor errors. However, I have a major concern about the presented results. LBD16 induction by salinity seems to be very specific and dependent on tissue and time (only one peak at 6 h of NaCl treatment in 4-week-old plants). Nevertheless, the experiments were performed with different aged plants mostly not specified in the figure legends and lacking coherence between ones and others. According to the**

Methods section, the phenotyping of LR was performed with 10-day-old seedlings (4 days grown in control conditions and 6 additional days for NaCl treatments). For LBD16::GFP expression patterns, authors used 6-day-old seedlings (plus 6 h treatment). For time course expression studies of LBD16 and ZAT6, they changed the temperature, the photoperiod, and the growing time (Col-0 plants were grown hydroponically under 12 h/12 h light/dark cycle, 20{degree sign}C until 4-week-old and then were treated with 0 mM or 125 mM NaCl for 0 h, 3 h, 6 h, 12 h, 18 h or 24 h.). For the RNA-Seq, they used 8-day-old seedlings and for cell wall analyses, plants grown in control conditions for 7 days and treated for 3 additional days. I do not understand such random sampling and think that this significantly diminishes the quality of the presented data, making hard their interpretation. I suggest homogenizing the analysis conditions to support their conclusions.
Major points:
LBD16 transcript levels, after salinity treatment, are reduced in the zat6 mutants (Figure 4b) but also the overexpressors (Figure 4c). The authors concluded that ZAT6 is a positive regulator of LBD16 in such conditions. I do not understand how this conclusion arose and why.
The regulation of LBD16 by ARF7 was already described (Orosa-Puente et al., 2018) and presented here as new information in the paragraph starting in line 171.
The induction of ZAT6 by NaCl was also already described before (Tang and Luo, 2018), as well as its relationship with osmotic stress (Liu et al., 2013). Moreover, osmotic stress and no NaCl toxicity could be generating the differential phenotype. This possibility should be tested. LBD16 expression in response to salinity was already investigated, and resulted in no response (Perotti et al., 2022); plants' age may explain the differences, but authors must discuss this point because is crucial for their work.
In normal growth conditions, LBD18 and LBD33 are redundant with LBD16 (line 133). What happens with these paralogs under salinity?
Authors show GFP expression in the primary root using plants transformed with LBD16genomic ::GFP, but after that, continued with all the studies in LR. Images of the same plants focused in LR should be shown.
Specific comments on illustrations
Figure 1 and S1:
Plants in panels 1a, b, and c, seem to be 7 or 8-day-old. However, the age of these plants is not mentioned in the legend. In Figure S1, it is described that 4-day-old plants were placed for 2-3 days in NaCl plates. I guess that the treatment and age were the same, but this must be mentioned.
LBD16 expression analyses were done in 4-week-old plants, according to the text. Why was expression analyzed in seedlings with different ages when it seems to be tissue and developmental stage-specific?
The differences signaled in the mock of Figure S2a are hard to visualize, too subtle.
1e, I understand that is a main root. This figure must be accompanied by the image of LR and the age of the plants, as stated in the legend.
Figure 2:
The authors stated in the text that LBD16 transcript levels increased in the double mutant arf7/19 (panel d). However, I cannot see this result in the corresponding illustration. LBD16 transcript levels appeared similar in Col-0 and double mutants with superposed error bars.
Again, the age of the plants is not signaled. From which tissue authors extracted the RNA? Whole seedlings? Whole roots? Please, explain.
In panel 2e: in the text, the authors referred to this panel talking about treatments for 3 or 6 hours of salt treatment. However, I cannot visualize two treatments or understand how they interpreted these results. I see only one bar corresponding to 125 mM and the control (0 mM).
S3: It is hard to see the differences in GUS staining between treated and untreated plants. How many independent lines did the authors use for this experiment?
Figure 4:
An empty vector is not a good control for this experiment. I suggest using an FT that not regulates LBD16 with the same promoter (35S).
Figure 5:
As it is shown, ZAT6 acts upstream of LBD16. However, in ZAT6 mutants there are not the same polysaccharide modifications observed in lbd16 knockout plants. Please, explain
Minor points
The internationally accepted nomenclature for "non-emerged lateral roots" is lateral root primordia (LRP) and for emerged ones, lateral roots (LR).
The authors used different forms to name proteins. For example LATERAL ORGAN BOUNDARY DOMAIN (LBD)16 and ZINC FINGER OF ARABIDOPSIS THALIANA 6 (ZAT6). In the first case, the number follows the abbreviated name, and in the second the complete name.
Modulate and modulation are repeated in the same sentence: converging on LBD16 to modulate root branching modulation under salinity.
Once the RSA abbreviation was introduced, authors continued to talk about "root system architecture".
In the sentence "Arabidopsis ARF7 and ARF19 were shown to mediate LR development by transcriptional regulation of several redundant LATERAL 61 ORGAN BOUNDARY DOMAIN (LBD)-type transcription factors including LBD16, 62 LBD18, LBD29 and LBD33 to mediate LR development", authors repeated, "mediate LR development".
In the paragraph starting in line 75, the authors described genes associated with LR development in response to

salinity stress. I find this paragraph incomplete; several other genes (including TFs) were associated with such response and modulate RSA. In particular, when they stated: "Nevertheless, these studies cannot explain how auxin signals are affected during LR development in salt, and how salt impacts the core LR developmental pathways is largely unknown.", they are ignoring significant contributions from other groups to this subject.
Line 99: is the encoded protein and not the gene the master regulator
Line 276: why the authors changed to 130 mM NaCl?

Reviewer #3 (Comments for the Author):

The manuscript, "Root branching in salt requires auxin-independent modulation of LBD16 function" by Zhang et al, is a very elegant study that shows that LBD16 can be an integrator of upstream signals for lateral root development. The Introduction is very well written providing the reader with all the information needed to understand the following experiments. The research objectives are clear and the experimental designed is very well-thought. I am totally convinced that *ZAT6* is a positive upstream regulator of LBD16 under salinity stress conditions and together they modulate downstream events for lateral root development in Arabidopsis.

Major Revision.
1.The only major comment concerns with the identification of the other putative upstream regulators under salt stress. Although, I understand the selection of *ZAT6* for pursuing the functional characterization, I do not think it is well explained why the other regulators were set aside. Namely, *WOX13* and *WRKY38* which bind directly *LBD16* promoter and in the author's network analysis under salt stress conditions were predicted to have a frequency between 90% to 99%. These are also very high frequencies. Thus, it would be interesting also to know the expression of *WOX13* and *WRKY38* in salt conditions and to search about their interaction with the *ARF7/19 - LBD16* path, likewise it was performed for *ZAT6*. This could provide a better insight into their involvement under this salt stress condition as well as their putative interactions with the lateral root development regulator modules, which could be further discussed in the appropriate section.

Minor Revisions.
1. The manuscript presents a high-level quality of images, except the ones in Figure S3. It might be because of the pdf provided but if not, I would advise to improve the quality of the root GUS staining in Figure S3a and Figure S3b.
2. The legends on images/ graphics can be sometimes very confusing. Specifically, in the graphics of Figure 4, there is an abuse of abbreviations that can be misleading: LRD (Lateral Root Development) or LRD (Lateral Root Density)?; What is NE (Non Emerged)?; What is ELR (Emerged Lateral Root)?. Thus, it should be NELR (Non Emerged Lateral Root). I advise a revision of legends on images/ graphics throughout the manuscript.
3. In line 44, the authors mention the "basal meristem". This definition was used just once throughout the manuscript (even in Figure 5f, it is not depicted). I would propose just to remove the word "basal" to be consistent.
4. In line 100, the authors mention the "Arabidopsis HapMap". This tool might be unknown outside the Arabidopsis research community. I would suggest explaining it briefly.
5. In line 236, please change *Nicotiana Benthamiana* to *Nicotiana benthamiana*.
6. Please revise the protocol nomenclature, sometimes the name of the gene is not in italic.

Dear Editors,

Thanks for your and all three reviewers' constructive and valuable comments on our manuscript. We have carefully read your comments and suggestions regarding the texts, figures and legends and the suggestions for additional experiments, and based on these we have provided additional clarifications and data in the manuscript to support our conclusions.
In particular
-We have performed additional qRT-PCR experiments using roots from seedlings of Col-0 and *arf7-1;arf19-3* mutants to assess the expression of LBD16 and ZAT6, and these data confirmed our earlier results obtained with roots from older plants. They also confirm our conclusion of salt-induced LBD16 expression in the *arf7/19* background.
-We tested a close homolog of ZAT6, ZAT10 as a control in the dual-luciferase assay in *Nicotiana benthamiana* leaves and found that only ZAT6 could activate the promoter activity of *LBD16*.
-In addition, by further data analysis of publicly available data we have clarified the expression domain of *ZAT6* in the roots upon salt stress.
-Finally we have improved figure legend descriptions to clarify age and treatment of seedlings.

We now submit a revised manuscript and our response letter addressing all points raised by the reviewers. Please find our point-to-point replies to the reviewers' comments in this letter.

We look forward to receiving your feedback on this revised version.

Best regards,
Yanxia Zhang and Christa Testerink, on behalf of all authors.

Reviewer #1:

Comment 1: The main element missing in the manuscript is in which tissues is LBD16 acting in response to salt treatment. LBD16 is classically reported to be involved in the early phase of LR development (esp. Stage 1); here it appears it also plays a role in a later phase. The effects of *lbd16* mutation are on emergence and salt triggers LBD16 expression in overlying tissues. I find these observations very interesting. Where is ZAT16 expressed? in overlying tissues? Are the cell wall modifications reported related to changes in the primordium and/or the overlying tissues?

Response 1: Thanks for this valuable point. Indeed, LBD16 was reported by Goh et al. (2012, Development) to be mainly expressed in the root pericycle cells of the main roots regulating early phase of LR formation. However, both Lee et al. (2009) and Lavenus et al. (2015) showed that pLBD16::GUS is also expressed throughout various stages of LR formation, not only in the main roots during early phase but also in the overlaying tissues during post-emergence, which is similar to what we observed for LBD16 in Figure S3C. Salt enhances LBD16 expression in the main root zones during early phases of LRP development as well as in the overlaying tissues of lateral root primordia(Fig.1 and Fig. S3B and C). In addition, Okushima et al. (2007) also reported that NAA treatment induces LBD16 expression in various root cell types, not only in the pericycle cells. All these indicate that LBD16 is not only acting in the pericycle cells during early phases of LR development. We have now clarified this the text starting from line 141 till line 146.

We have further clarified the expression domain of ZAT6 in comparison with LBD16 expression under control and salt conditions in Figure S5C.
As shown by current Figure S5C, according to Geng et al., 2013, the expression pattern of ZAT6 is highly consistent with LBD16 in the root cell layers involved in LR development upon salt stress, in particular in the root stele layer. In addition, ZAT6 was shown to be enriched in the xylem pole pericycle cells and involved in Na^+ ion allocation between roots and shoots by Fabado et al. 2022 (https://www.biorxiv.org/content/10.1101/2022.02.04.479129v1).

Regarding the salt-induced cell wall modifications, we have further clarified in the text in the 2nd and 3rd paragraphs of the Discussion. Wachsman et al. (2020, Science) and Zhu et al. (2019, Nature Plants) have shown that cell wall modifications are required for both LR initiation in the main root zones and the LRP development during the lateral root outgrowth. Therefore, LBD16-mediated cell wall modifications are likely occurring in both main root zones and the overlaying tissues of LRP.

Comment 2: Corollary to this, where is _LBD16_ expressed in response to salt treatment in the in the _ZAT16_ mutants (loss of function or _ZAT16-SRDX_)?

Response 2: Thanks for this very good point. We did make a transgenic line carrying proLBD16::GUS in *zat6-1* mutant background and checked its GUS expression, but we did not see any GUS signals in this line under control condition. We assume the GUS signal was silenced in the mutant background, so we did not follow up with this line, and as such we cannot directly answer this question. However, as our qRT-PCR data for LBD16 expression in *zat6-1, zat6-2* and the repressor line 35S::ZAT6::SRDX backgrounds are consistent with each other, we are confident about the regulation of *LBD16* being dependent on ZAT6 in response to salt.

Comment 3: A second point is related to Fig 2D. The authors write "after 6 h salt treatment LBD16 expression was increased in the arf7-1;arf19-3 double mutant compared to Col-0". Yet, only a weak increase is visible on the graph and it does not appear to be different from the null hypothesis. The wording should be adapted. This key result is further confirmed with the ARF7-GR in arf7/19 background, so I do not doubt its validity, I am however surprised that the increase is much more marked (2E, Mock) than in arf7-1;arf19-3. Why that?

Response 3: Thanks for this remark. We realized that we did not phrase this well in the text. We have performed an additional experiment with 7-day-old seedlings (we previously used 5-day-old seedlings; see also our response to Rev 2) of Col-0 and *arf7-1;arf19-3* mutants in control and salt conditions at 0 h, 3 h, 6 h, 12 h, 24 h and 30 h, and confirmed that LBD16 expression was increased in *arf7-1;arf19-3* double mutant compared to Col-0 starting from 6 h salt treatment and lasting till 24 h. We have revised the text line accordingly.
Regarding the differences in the LBD16 expression between Fig.2D and 2E, we have replaced figure 2D with new data from 7-day-old seedlings of both Col-0 and *arf7-1;arf19-3* mutants and data are consistent between 2D and 2E, showing that salt-induced LBD16 expression occurs in both *arf7-1;arf19-1* and *arf7-1;arf19-3* double mutants.

Minor comments:
-l101: double "identified"
-l154: missing call to supp figure (Fig. 1c and Fig. Sc)
Response 4: Thank you. we have revised the texts accordingly.

Reviewer #2

Comment 1: I have a major concern about the presented results. LBD16 induction by salinity seems to be very specific and dependent on tissue and time (only one peak at 6 h of NaCl treatment in 4-week-old plants). Nevertheless, the experiments were performed with different aged plants mostly not specified in the figure legends and lacking coherence between ones and others. According to the Methods section, the phenotyping of LR was performed with 10-day-old seedlings (4 days grown in control conditions and 6 additional days for NaCl treatments). For LBD16::GFP expression patterns, authors used 6-day-old seedlings (plus 6 h treatment). For time course expression studies of LBD16 and ZAT6, they changed the temperature, the photoperiod, and the growing time (Col-0 plants were grown hydroponically under 12 h/12 h light/dark cycle, 20{degree sign}C until 4-week-old and then were treated with 0 mM or 125 mM NaCl for 0 h, 3 h, 6 h, 12 h, 18 h or 24 h.). For the RNA-Seq, they used 8-day-old seedlings and for cell wall analyses, plants grown in control conditions for 7 days and treated for 3 additional days. I do not understand such random sampling and think that this significantly diminishes the quality of the presented data, making hard their interpretation. I suggest homogenizing the analysis conditions to support their conclusions.
Response 5: Thanks for raising this point. We agree and have now specified the plant age and treatment time in each figure legend.
Moreover, regarding the point using hydroponically grown roots, we have performed additional experiments to study the time course expression of *LBD16* in 7-day old seedlings of Col-0 and *arf7-1;arf19-3* mutants treated with salt for 0 h, 3 h, 6 h, 12 h, 24 h and 30 h under long-day condition, and confirmed its expression patterns are similar to what we observed using 4-week old plants grown hydroponically at mid-day conditions. Accordingly, we have moved the time course expression data from roots of 4-week- old plants to supplementary data as Figure S3A.
For the ages of seedlings used for phenotyping vs gene expression, since we assessed the LR phenotypes using 10-day old seedlings, so we analyzed the gene expression before 10 days, and at a stage when the roots contain all different stages of LRP and emerged LRs, for which we considered 6~8-day old is the best under our growth condition. For the gLBD16::GFP expression, the reason we used 6-day-old seedlings is that under our growth condition 6-day-old is best stage to observe the salt-

response of main root and small LRs, since the seedlings starting to have a few small emerging LRs, consisting with the proLBD16::GUS activity experiment in Figure S3B-C.
For the cell wall analysis, we measured at two timepoints, 7-day old + 24 h salt treatment and 7-day old + 72 h salt treatment (Figure 5D and Figure S10) as we expected that cell wall sugar changes are the consequences of cell wall remodeling gene expression.
For the RNA-seq experiment, the reason we used 8-day-old seedlings was partially due to this experiment being part of a bigger experimental design to be consistent with other projects in the lab. We considered 8-day is also a good stage to access gene expression since the seedlings are in a fast-growing stage in terms of main root derived LRs.

Comment 2: LBD16 transcript levels, after salinity treatment, are reduced in the zat6 mutants (Figure 4b) but also the overexpressors (Figure 4c). The authors concluded that ZAT6 is a positive regulator of LBD16 in such conditions. I do not understand how this conclusion arose and why.
Response 6: The *35S::ZAT6::SRDX* line used in Figure 4C is not a regular OE line, but a constitutive repression line of ZAT6. With the synthetic SRDX repression domain, the transcription of ZAT6 in the 35S::ZAT6::SRDX line is suppressed, thus the expression of LBD16 in this line is expected to be similar to that of the *zat6* T-DNA lines. As lack of ZAT6 retards LBD16 expression, hence we concluded that ZAT6 is a positive regulator required for LBD16 function under salt. To avoid confusion, we have added "repressor" to the description of the line in Materials and Methods, Results and Figure legend.
Comment 3: The regulation of LBD16 by ARF7 was already described (Orosa-Puente et al., 2018) and presented here as new information in the paragraph starting in line 171.
The induction of ZAT6 by NaCl was also already described before (Tang and Luo, 2018), as well as its relationship with osmotic stress (Liu et al., 2013). Moreover, osmotic stress and no NaCl toxicity could be generating the differential phenotype. This possibility should be tested. LBD16 expression in response to salinity was already investigated, and resulted in no response (Perotti et al., 2022); plants' age may explain the differences, but authors must discuss this point because is crucial for their work.

Response 7: Thank you for these relevant and detailed comments. Regarding the previous studies on LBD16 and ZAT6, we have rephrased the text (line 176 to 181) and agree that part of our data in Figure 2E is confirming the previously published results. In addition, we agree that ZAT6 was already linked to salt and osmotic stress before. Though these findings were not linking LBD16 and ZAT6 in regulating LR development under salt stress, which is shown in our present manuscript, they are supporting the role of the identified ZAT6-LBD16 module in salt stress responses.
Regarding the composition of salt stress, indeed, NaCl treatment generates both ionic and osmotic stresses. It is of course interesting to know whether the phenotype of *lbd16* mutants is salt-specific or not. However, as we mentioned in the manuscript, we started to assess LBD16 function in salt stress since it was found in one of the candidate loci mapped for root system architecture in response to salt stress. Therefore, we continued the research using salt conditions only.
Indeed, Perotti et al., 2022 showed pLBD16-GUS has no salt response, but they used 3-day old plants and treated with 75mM NaCl for 5 days, which might be too long to capture the salt-induced LBD16 expression peak. According to our data, salt (125mM NaCl)-induced LBD16 expression is fluctuating, and usually peaked between 6 h to 12 h depending on the plant age.

Comment 4: In normal growth conditions, LBD18 and LBD33 are redundant with LBD16 (line 133). What happens with these paralogs under salinity?
Response 8: Thanks for this remark. We agree that LBD proteins might have functional redundancy according to previous publications, and we might not rule out the role of LBD18 or LBD33 during salt response. However, these are not the scope of this study.

As mentioned in Response 7, we started this study since LBD16 was identified by a GWAS study to be involved in root system architecture regulation in response to salt. We further confirmed the salt-induced phenotype of lbd16 mutants and this reduced LR density phenotype could be complemented by LBD16 in gLBD16 line (Figure 1). Hence, we propose this phenotype is caused by LBD16, not LBD18 or LBD33.

Comment 5: Authors show GFP expression in the primary root using plants transformed with LBD16genomic ::GFP, but after that, continued with all the studies in LR. Images of the same plants focused in LR should be shown.

Response 9: Given the fact that LBD16 is expressed during various stages of LRP development in the primary roots before LR emergence (Figure 1D and Figure S3B and C) (Lavenus et al., 2015), we showed gLBD16-GFP expression in the primary roots. We also tried to zoom into the LRP to image gLBD16-GFP, but we could not quantify the expression well. Therefore, we decided to show the overall LBD16 expression in the main root zones which contain different stages of LRP.

Comment6: Figure 1 and S1: Plants in panels 1a, b, and c, seem to be 7 or 8-day-old. However, the age of these plants is not mentioned in the legend. In Figure S1, it is described that 4-day-old plants were placed for 2-3 days in NaCl plates. I guess that the treatment and age were the same, but this must be mentioned.
Response 10: Thank you, this has now been clarified in the figure legends.

Comment7: LBD16 expression analyses were done in 4-week-old plants, according to the text. Why was expression analyzed in seedlings with different ages when it seems to be tissue and developmental stage-specific?
Response 11: Thanks for this remark. We have provided additional data on LBD16 expression in response to salt in Figure 2D by using 7-day-olding seedling of Col-0 and *arf7-1;arf19-3* double mutants after treatment with salt for 0 h, 3 h, 6 h, 12 h, 24 h and 30 h, making sure that all the qRT-PCR data in the main figures used 7-day-old seedling.
Please also see Response 5 for Ref#1.

Comment8: The differences signaled in the mock of Figure S2a are hard to visualize, too subtle. 1e, I understand that is a main root. This figure must be accompanied by the image of LR and the age of the plants, as stated in the legend.
Response 12: The difference signaled under mock in Figure S2A is for main root under control condition which is not the central point of this figure. The key point we want to address with this Figure S2A and 2B together is that the LR density difference between Col-0 and lbd16 mutants under salt stress is not caused by the difference in main root length of these two genotypes. We have clarified the plant age in the figure legends of Figure S2.

Comment 9: Figure 2: The authors stated in the text that LBD16 transcript levels increased in the double mutant arf7/19 (panel d). However, I cannot see this result in the corresponding illustration. LBD16 transcript levels appeared similar in Col-0 and double mutants with superposed error bars. Again, the age of the plants is not signaled. From which tissue authors extracted the RNA? Whole seedlings? Whole roots? Please, explain.
Response 13: Thank you, this was not phrased clearly. We have revised the text to clarify and improved the figure illustration, and specified the information of the samples in the figure legends. Also 2D was replaced with additional gene expression data with more timepoints (see also Response 5 to Ref #1)

Comment 10: In panel 2e: in the text, the authors referred to this panel talking about treatments for 3 or 6 hours of salt treatment. However, I cannot visualize two treatments or understand how they interpreted these results. I see only one bar corresponding to 125 mM and the control (0 mM).
Response 14: We have revised Figure 2E with the information for treatment time in the figure title.

Comment 11: S3: It is hard to see the differences in GUS staining between treated and untreated plants. How many independent lines did the authors use for this experiment?
Response 15: Figure S3B and C represent data from two independent transgenic lines from at least 3 independent experiments. We have clarified this better in the figure legends.

Comment 12: Figure 4:
An empty vector is not a good control for this experiment. I suggest using an FT that not regulates LBD16 with the same promoter (35S).

Response 16: We have now provided ZAT10 as a control in current Figure S6A. ZAT10 is a close homolog of ZAT6 (Xie et al., 2019), but it is not regulating LBD16 promoter activity in our promoter activity assay.

Comment 13: Figure 5:
As it is shown, ZAT6 acts upstream of LBD16. However, in ZAT6 mutants there are not the same polysaccharide modifications observed in lbd16 knockout plants. Please, explain

Response 17: Indeed, though *zat6* mutants and *lbd16* mutants showed similar degree of methylation in cell wall polysaccharides we did not observe exactly the same cell wall modification patterns in these mutants. ZAT6 may only have a milder cell wall phenotype.

Comment 14: The internationally accepted nomenclature for "non-emerged lateral roots" is lateral root primordia (LRP) and for emerged ones, lateral roots (LR).
Response 18: We have revised the figure, text and figure legends accordingly. Now ELR represents emerged lateral roots, and LRP represents non-emerged lateral root primordia.

Comment 15: The authors used different forms to name proteins. For example LATERAL ORGAN BOUNDARY DOMAIN (LBD)16 and ZINC FINGER OF ARABIDOPSIS THALIANA 6 (ZAT6). In the first case, the number follows the abbreviated name, and in the second the complete name.
Response 17: Since LBD16 and ZAT6 are not newly identified proteins in our manuscript, we followed the previous publications for their abbreviations to avoid confusions.

Comment 16:
-Modulate and modulation are repeated in the same sentence: converging on LBD16 to modulate root branching modulation under salinity.
-Once the RSA abbreviation was introduced, authors continued to talk about "root system architecture".
-In the sentence "Arabidopsis ARF7 and ARF19 were shown to mediate LR development by transcriptional regulation of several redundant LATERAL 61 ORGAN BOUNDARY DOMAIN

(LBD)-type transcription factors including LBD16, 62 LBD18, LBD29 and LBD33 to mediate LR development", authors repeated, "mediate LR development".

-In the paragraph starting in line 75, the authors described genes associated with LR development in response to salinity stress. I find this paragraph incomplete; several other genes (including TFs) were associated with such response and modulate RSA. In particular, when they stated: "Nevertheless, these studies cannot explain how auxin signals are affected during LR development in salt, and how salt impacts the core LR developmental pathways is largely unknown.", they are ignoring significant contributions from other groups to this subject.

-Line 99: is the encoded protein and not the gene the master regulator

Response 18: Thank you, all of these issues have been revised in the text accordingly.

Regarding the literatures on how salt impacts core LR developmental pathways, it is indeed not much reported. After critical selection, we have now cited more previous studies regarding LR development in response to salt in the introduction, including Zhao et al., 2011 on SOS3 regulating LR development under salt, and WRKY46 (Ding et al., 2015) involved in LR development via auxin pathway under salt.

Comment 16: Line 276: why the authors changed to 130 mM NaCl?

Response 19: To be able to cut the root samples precisely after salt treatments for RNA-seq analysis, we grew the Arabidopsis seedlings on a very thin layer of square-shape nylon mesh, which prevents the direct contacts of the roots with the salty medium. To compensate the dilution of this thin layer of nylon mesh on the expected salt concentrations, we decided to use 130mM NaCl instead of 125mM.

Reviewer #3 :

Comment 1: The only major comment concerns with the identification of the other putative upstream regulators under salt stress. Although, I understand the selection of ZAT6 for pursuing the functional characterization, I do not think it is well explained why the other regulators were set aside. Namely, WOX13 and WRKY38 which bind directly LBD16 promoter and in the author's network analysis under salt stress conditions were predicted to have a frequency between 90% to 99%. These are also very high frequencies. Thus, it would be interesting also to know the expression of WOX13 and WRKY38 in salt conditions and to search about their interaction with the ARF7/19 - LBD16 path, likewise it was performed for ZAT6. This could provide a better insight into their involvement under this salt stress condition as well as their putative interactions with the lateral root development regulator modules, which could be further discussed in the appropriate section.

Response 20: Thanks for raising this valuable point. Indeed, according to the network inference, the candidate genes with frequency between 90% to 99% are also worthy to be further investigated, similar to ZAT6. These will indeed be the focus of future projects in the group though they are not within the scope of this manuscript.

For example, WOX13 has already popped up in the study performed by Vanneste et al.,2005 regarding LR initiation (https://academic.oup.com/plcell/article/17/11/3035/6114691).

DIV2 is a MYB protein that has been shown to play a negative role during salt stress (Fang et al., 2018, https://link.springer.com/article/10.1007/s00299-018-2321-6) and is required for ABA signaling (Song et al., 2016, https://www.science.org/doi/10.1126/science.aag1550).

This information also supports our results on network inference for LR developmental genes in response to salt stress.

Comment 2: The manuscript presents a high-level quality of images, except the ones in Figure S3. It might be because of the pdf provided but if not, I would advise to improve the quality of the root GUS staining in Figure S3a and Figure S3b.
Response 21: Thank you, we agree. We have replaced the GUS staining data with high quality images.

Comment 3: The legends on images/ graphics can be sometimes very confusing. Specifically, in the graphics of Figure 4, there is an abuse of abbreviations that can be misleading: LRD (Lateral Root Development) or LRD (Lateral Root Density)?; What is NE (Non Emerged)?; What is ELR (Emerged Lateral Root)?. Thus, it should be NELR (Non Emerged Lateral Root). I advise a revision of legends on images/ graphics throughout the manuscript.
Response 21: Thanks for this suggestion. We have revised the abbreviations in the graphs, texts and figure legends accordingly.

Comment 4:
-In line 44, the authors mention the "basal meristem". This definition was used just once throughout the manuscript (even in Figure 5f, it is not depicted). I would propose just to remove the word "basal" to be consistent.
-In line 100, the authors mention the "Arabidopsis HapMap". This tool might be unknown outside the Arabidopsis research community. I would suggest explaining it briefly.
-In line 236, please change Nicotiana Benthamiana to Nicotiana benthamiana.
-Please revise the protocol nomenclature, sometimes the name of the gene is not in italic.
Response 22: Thanks for these very useful comments to improve our manuscript. We have revised the text accordingly.

1st Revision - Editorial Decision 07-Oct-2023

Dear Dr. Christa Testerink:

We have received reviews of your manuscript entitled "Root branching in salt requires auxin-independent modulation of LBD16 function." On the basis of the advice received, the board of reviewing editors would like to accept your manuscript for publication in The Plant Cell. This acceptance is contingent on revision based on the comments of our reviewers. In particular, please consider the following:

Reviewer 2 still raises a valid and relevant point that the authors do not provide any evidence of whether the LBD16 phenotypes they observe are specific to the sodium effect of salt stress or are responsive to the osmotic component of the stress. While we do not expect the authors to do experiments with other stress treatments at this point, the authors should clearly acknowledge this uncertainty in the manuscript. This point could readily be added to the discussion.

The authors also should carefully go through the resubmission checklist and be sure that all instructions therein are adhered to. Particularly, it is asked that individual data points should be shown in all cases where n is less than six (and is strongly encouraged in other cases also). This will require reformatting a number of figures in the manuscript.

Please highlight all changes and include a detailed annotation of changes of the text, with line numbers, and noting your responses to the comments.

To submit your revised manuscript, click:

Link Not Available

If you have any questions about the revision submission procedures, please contact the Editorial Office Staff (tpc-submissions@aspb.org). If you cannot return the revised manuscript within 30 days, please let us know. Otherwise, we will assume that you have elected not to revise the manuscript and withdraw it.

Thank you very much for the privilege of reviewing this work. I look forward to receiving the next version.

Sincerely,

The Plant Cell Editorial Board

Reviewer #1 (Comments for the Author):

I thank the authors for their detailed answers to all the point I raised and providing additional data. The changes in the revised manuscript are addressing all the points I had.

Reviewer #2 (Comments for the Author):

The authors addressed several of the criticisms presented, particularly the one concerning the different plant ages at which the experiments were done. However, most answers were "this was not the scope of the work"; this kind of answer is not satisfactory for this reviewer.
This is submission to The Plant Cell, one of the most prestigious journals in the discipline. In my opinion, all the requested controls are needed.
Response 1: the authors confirmed the expression induction in 7-day-old seedlings and explain the varied sampling of roots to perform the different assays. However, as I told in the first revision, samples were taken with different aged plants treated with varied NaCl concentrations during different periods. They answer in comment 3: "According to our data, salt (125mM NaCl)-induced LBD16 expression is fluctuating, and usually peaked between 6 h to 12 h depending on the plant age." This is contradictory with the previous answer.
Response 4: The authors answered "these are not the scope of this study", and "Hence, we propose this phenotype is caused by LBD16, not LBD18 or LBD33." It is not difficult to test this instead of propose that the redundant genes are not involved in this response because LBD16 rescued the phenotype.

Response 5: In Figure 1D, the authors stated that there is an induction of LBD16 expression after the treatment with 150 mM

NaCl. I cannot see such induction and do not understand how the quantification shown in Figure 1E was done.
Response 7: I asked to differentiate if the differential phenotype observed was due to salinity or osmotic stress. The answer: "... started to assess LBD16 function in salt stress since it was found in one of the candidate loci mapped for root system architecture in response to salt stress. Therefore, we continued the research using salt conditions only...." I can understand that they continue the study with salt only, but the term "salt stress" is even in the title of the manuscript. Hence, they must be sure that is indeed NaCl and not the osmotic pressure causing the effect. It is not so difficult to test this.
Comment 14: The suggestion was to name, as all the literature about root architecture and development, emerged lateral roots as LR, and not ELR.
In Figure S3, LR stages were not shown as claimed.

Comment 8: the answer to my comment: "The differences signaled in the mock of Figure S2a are hard to visualize" was "...which is not the central point of this figure". If it is not important, Why it is signaled this as a result?

Reviewer #3 (Comments for the Author):

All my previous remarks were addressed. I do not have any further comments. Thank you!

Please also note the following:

-The Plant Cell now requires authors to complete and submit an author revisions checklist upon submission of a revised manuscript. The aim of the checklist is to aid authors in preparing a high-quality manuscript, facilitate the review and assessment of revised manuscripts, and help to ensure that journal standards are maintained across the board. If your manuscript is accepted, the completed checklist will be published as supplemental material attached to the article online. Please download a copy of the checklist (pdf fillable form) at this link, for submission with your revised manuscript: https://tpc.msubmit.net/html/Author_Revisions_Checklist.pdf.

-Supplemental materials should be restricted to large datasets and tables, presentation of replicates, and validation of reagents, methods, or genotypes. Any data that are used to support the major claims must be in the main manuscript. Supplemental figure legends must indicate what figure in the main manuscript is supported by the supplemental data presented. Please justify how each of the supplemental figures meet the criteria.

-Sampling methods and the nature of "biological replicates" should be described precisely (i.e. different plants, parts of plants, pooled tissue, independent pools of tissue, sampled at different times, etc.), along with a clear description of and rationale for any statistical analyses conducted. The reader should know exactly what was sampled; what forms the basis of the calculation of any means and statistical parameters reported. This is also necessary to ensure that proper statistical analysis was conducted.

Dear editors,

We sincerely thank the editorial board and all the three referees for their time and additional comments on our manuscript. We have carefully read all the comments, and provided point-to-point replies to the comments of reviewer 2.
Accordingly, we have revised the text to provide additional clarifications.

In addition, we also have gone through the resubmission checklist and made sure that all instructions therein were adhered to. We have re-made all the figures where individual data points are shown where n is less than six. For the figures (Figure 2C, 2D and Figure 3D) with multiple treatments and multiple timepoints where the data were not appropriate to show as individual data points, we have provided the raw data in new supplemental dataset 5.

Now we resubmit the revised version of the manuscript with a clean version and a track changes version. We think that the current manuscript has addressed all the issues raised by the editorial board and reviewers and look forward to hearing your feedback on this revised version.

Best regards,
Yanxia Zhang and Christa Testerink, on behalf of all authors.

Reviewer #2:

Comment 1: The authors addressed several of the criticisms presented, particularly the one concerning the different plant ages at which the experiments were done. However, most answers were "this was not the scope of the work"; this kind of answer is not satisfactory for this reviewer. This is submission to The Plant Cell, one of the most prestigious journals in the discipline. In my opinion, all the requested controls are needed.
Response 1: the authors confirmed the expression induction in 7-day-old seedlings and explain the varied sampling of roots to perform the different assays. However, as I told in the first revision, samples were taken with different aged plants treated with varied NaCl concentrations during different periods. They answer in comment 3: "According to our data, salt (125mM NaCl)-induced LBD16 expression is fluctuating, and usually peaked between 6 h to 12 h depending on the plant age." This is contradictory with the previous answer. Response 4: The authors answered "these are not the scope of this study", and "Hence, we propose this phenotype is caused by LBD16, not LBD18 or LBD33." It is not difficult to test this instead of propose that the redundant genes are not involved in this response because LBD16 rescued the phenotype.

Response 1: We thank this reviewer and the other two reviewers for all comments that helped us to improve the manuscript. We addressed more than 20 responses from all three reviewers with additional experiments and/or textual and figure corrections and additions, though indeed for 2 cases we felt the issue was outside the scope of the manuscript. One is Comment4 from Ref 2, about the LBD18 and LBD33. We have now provided additional clarifications and citations regarding the possible role of other LBD16-reduendent LOBs during salt-regulated root branching in the discussion, line 391 to line 401.

The other case we felt outside the scope is comment 1 raised by Reviewer#3, which is about the other candidates (DIV2, WOX13 and WRKY38) prioritized by the network inference. In fact, the functional characterizations of these genes during rooting in salt are on-going works in our group. We have now included textual discussions about their possible roles during plant salt stress response or root branching, line 435 to line 440.

Comment 2: Response 5: In Figure 1D, the authors stated that there is an induction of LBD16 expression after the treatment with 150 mM NaCl. I cannot see such induction and do not understand how the quantification shown in Figure 1E was done.

Response 2: Thanks for this point. As described in the Figure 1 legend, we have used 125mM NaCl (not 150 mM) to treat the roots of gLBD16-GFP plants, and we observe more GFP signal in the elongation and differentiation zones (indicated by yellow arrows in current Figure 1D). The method for GFP intensity quantification has been further clarified in the material and method section, line 599 to 603.

Comment 3: Response 7: I asked to differentiate if the differential phenotype observed was due to salinity or osmotic stress. The answer: "... started to assess LBD16 function in salt stress since it was found in one of the candidate loci mapped for root system architecture in response to salt stress. Therefore, we continued the research using salt conditions only...." I can understand that they continue the study with salt only, but the term "salt stress" is even

in the title of the manuscript. Hence, they must be sure that is indeed NaCl and not the osmotic pressure causing the effect. It is not so difficult to test this.

Response 3: Thanks for this point. Indeed, we are aware and totally agree that NaCl-induced salt stress consists of both osmotic stress and ionic stress. Since we continued the research under salt treatment only, we did not emphasize NaCl /ionic/osmotic stress specificity, so the osmotic component of salt stress could also contribute to this phenotype. We did **not use salt stress** in the title, but only “salt”. In response to this comment, for clarity, we have changed the word “salinity” to “ salt” where necessary in the text and have also further discussed that this phenotype might be not salt/NaCl-specific in the discussion, line 456 to line 458.

Comment 4: Comment 14: The suggestion was to name, as all the literature about root architecture and development, emerged lateral roots as LR, and not ELR.
In Figure S3, LR stages were not shown as claimed.

Response 4: Thanks for this point. We have revised the names in the text, figure and figure legends.

Comment 5: Comment 8: the answer to my comment: "The differences signaled in the mock of Figure S2a are hard to visualize" was "...which is not the central point of this figure". If it is not important, Why it is signaled this as a result?

Response 5: Thanks for this point. We should have clarified this better in the response letter. What we wanted to address with Figure S2A is that under salt conditions there were no differences in the main root length among Col-0, lbd16-1 and gLBD16 lines (the light and dark blue bars), whereas there was reduced LR density in *lbd16-1* (Figure S2B), indicating that the salt-induced LR phenotype in *lbd16-1* is not due to changes in the main root length. The differences in main root length are statistically significant in the mock treatment (black bars), but not under both salt treatments.

Dear Dr. Christa Testerink:

We are pleased to inform you that your paper entitled "Root branching in salt requires auxin-independent modulation of LBD16 function" has been accepted for publication in The Plant Cell, pending a final editorial review by a science editor. At this stage, your manuscript will be evaluated by a science editor with respect to its presentation of scientific content, compliance with journal policies, and presentation for a broad readership. The Plant Cell has contracted with Plant Editors (planteditors.com) to provide this service to our authors, and you will soon receive additional information on this process.

Please note that each author needs to link their ORCID identifier to their account in the system before your manuscript can be published. If any authors do not have an ORCID linked to their account, they will receive a message with a link to complete this task. Please ensure that ALL of your coauthors have completed this task as soon as possible.

ASPB offers an OPEN option that allows authors to have their online articles available for free to all users immediately upon publication. For more information about the ASPB OPEN option, refer to the Final Submission Checklist Form.

The Plant Cell and The Arabidopsis Information Resource (TAIR) are collaborating to collect functional annotation data about Arabidopsis genes from authors. This includes information about the gene's molecular function (e.g., kinase activity, ATP synthetase activity), the biological process/es it is involved in (e.g., endosperm development, threonine biosynthesis), its subcellular location (e.g., nucleus, ER), anatomical or developmental expression pattern (e.g., leaf, ovule, flower stage 10, seedling stage), or its partner in a protein-protein interaction (e.g., AT1G01010 interacts with AT1G01020).

If your paper contains results falling into one or more of these categories for Arabidopsis genes, we request that you now submit these data for inclusion in TAIR by filling in the form provided at the following URL: https://www.arabidopsis.org/doc/submit/functional_annotation/123. If you need further clarification on what types of data can be submitted please contact curator@arabidopsis.org.

Finally, we encourage your submission of artwork for the journal cover. Monthly issues will have an online cover image and selected covers will be used for posters, other promotional items, and "wallpaper" for mobile devices. For more information, refer to Cover Submission in the Instructions for Authors https://tpc.msubmit.net/cgi-bin/main.plex?form_type=display_auth_instructions.

We look forward to seeing your paper published.

Sincerely,

The Plant Cell Editoral Board

=====
IMPORTANT REMINDER: PEER REVIEW REPORTS
=====

If you opted to publish a peer review report along with your article during the original submission process, it will be prepared by the editorial staff and publicly posted with your manuscript, inside the zip file that contains any other supplemental material. As a reminder, the peer review report is a public record of all comments from editors and reviewers, as well as your prior responses, as you received them in the decision letters for each draft of your manuscript. If you agreed to publish this report and have changed your mind, or are not sure if you selected this option, please contact the editorial office as soon as possible before signing the license agreement from our publisher.

=====
REVIEWER COMMENTS
=====

----FOR ASPB OFFICE USE ONLY (DO NOT EDIT)----
MSID: 34476
Corresponding Author: Prof. Dr. Testerink
